# Bridging Scales: Integrating Hyaluronan-Based Nanoencapsulation into 3D Hydrogel Constructs for Advanced Therapeutics—A Comprehensive Review

**DOI:** 10.3390/gels12090780

**Published:** 2026-09-01

**Authors:** Mohamed E. Hassan

**Affiliations:** Group of Advanced Materials and Nanotechnology, Centre of Scientific Excellence, Chemistry of Natural and Microbial Products Department, National Research Centre, El Behouth Street, Cairo 12622, Egypt; mohassan81@gmail.com or me.hassan@nrc.sci.eg; Tel.: +20-11-1788-7429

**Keywords:** drug delivery, tissue engineering, regenerative medicine, CD44 (cluster of differentiation 44) targeting, bioprinting

## Abstract

The integration of nanoscale drug carriers within macroscale three-dimensional (3D) hydrogel architectures represents a paradigm shift in biomaterials design, enabling hierarchical control over therapeutic delivery. Hyaluronan (hyaluronic acid, HA), a native glycosaminoglycan of the extracellular matrix (ECM), has emerged as an exceptionally versatile building block for both nanoscale encapsulation systems and macroscale hydrogel scaffolds. This comprehensive review examines the rationale, fabrication strategies, and therapeutic applications of hyaluronan-based nanoencapsulation integrated within 3D hydrogel constructs, highlighting how this multiscale approach bridges the gap between molecular drug stabilization and tissue-level regenerative outcomes. We explore the chemical versatility of HA, the design principles of nanoencapsulation systems, the synthesis of 3D HA hydrogels, and the strategies for integrating these systems into unified therapeutic platforms. Applications in 3D bioprinting, injectable hydrogels, organ-on-a-chip models, cancer therapy, and tissue regeneration are critically evaluated. Finally, we discuss translational challenges, regulatory considerations, and future perspectives for clinical implementation of these advanced therapeutic systems.

## 1. Introduction: The Multiscale Therapeutic Challenge

Traditional drug delivery systems often operate at a single length scale, limiting their ability to address the complex spatiotemporal requirements of tissue regeneration and disease treatment. Free therapeutics suffer from rapid clearance, poor bioavailability, and off-target effects, while simple hydrogel depots frequently exhibit burst release kinetics and limited control over drug stability. The convergence of nanotechnology and three-dimensional (3D) hydrogel engineering offers a solution: nanoencapsulation within 3D hydrogel constructs creates a hierarchical delivery platform where nanoparticles (NPs) provide molecular-level protection and controlled release, while the hydrogel matrix provides tissue-level structural support, injectability, and prolonged residence time [1,2].

The convergence of nanotechnology and hydrogel engineering represents a paradigm shift in the development of advanced therapeutic platforms. Hyaluronan (HA), a naturally occurring glycosaminoglycan prevalent in pericellular matrices, body fluids, and specialized tissues, has emerged as a cornerstone material in both nanomedicine and hydrogel engineering due to its exceptional biocompatibility, biodegradability, and unique receptor-mediated targeting capabilities [3]. The structural composition of HA, featuring alternating units of D-glucuronic acid and N-acetyl-D-glucosamine connected by β-(1→3) and β-(1→4) glycosidic linkages, endows it with remarkable physicochemical properties including high water-binding capacity, viscoelasticity, and tunable mechanical characteristics [4].

**Figure 1:**
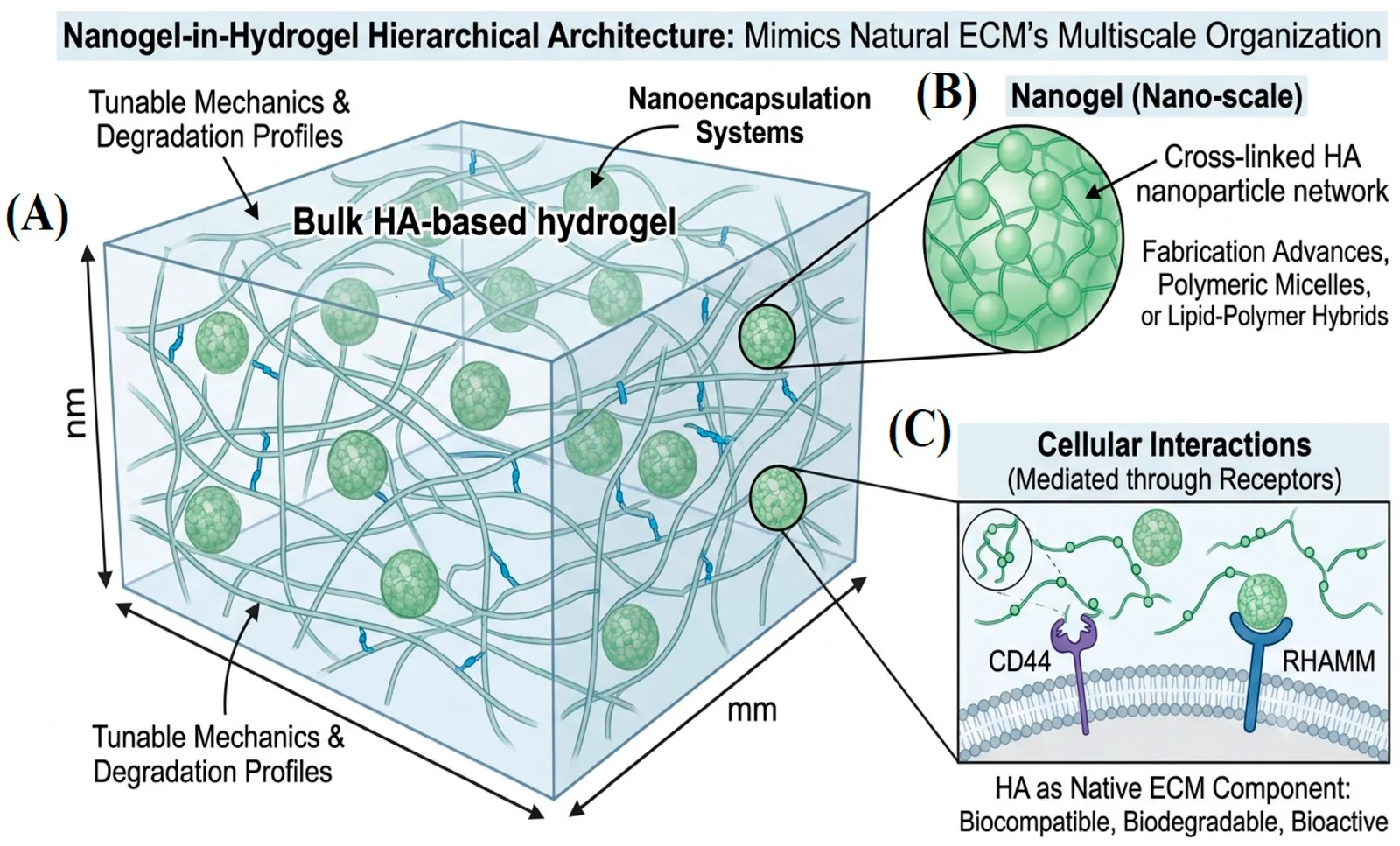
Schematic overview of the hyaluronan (HA)-based “nanogel-in-hydrogel” hierarchical architecture. (**A**) Representative 3D illustration of a bulk HA-hydrogel construct (macro-scale, mm-range) with embedded HA-nanogels (nano-scale). Key features, such as tunable mechanics and degradation profiles, are highlighted. Scale bars denote the macro (mm) and nano (nm) domains. (**B**) Magnified inset detailing the cross-linked HA nanoparticle network within a single nanogel (50–300 nm). (**C**) Schematic representation of the cellular interactions and native biological activity mediated by HA. Magnified view shows specific recognition of HA by CD44 and RHAMM receptors on a cell membrane, illustrating the material’s biocompatibility, biodegradability, and inherent bioactivity. Figure concept was developed using Google Gemini (3.7 Flash) for initial schematic suggestions, followed by extensive author editing and refinement based on concepts derived from the cited literature. No AI-generated images were used directly; the final figures were prepared by the author. For AI usage disclosure, see the statement at the end of the manuscript.

Hyaluronan is uniquely positioned for this dual-scale application. As a native ECM component, HA is inherently biocompatible, biodegradable, and bioactive, with its biological effects mediated through receptors such as CD44 and RHAMM. Recent advances have enabled the fabrication of HA-based nanogels, polymeric micelles, and lipid-polymer hybrid nanoparticles, as well as chemically modified HA hydrogels with tunable mechanics and degradation profiles [5]. Integrating these nanoencapsulation systems within 3D HA hydrogels creates a “gel-in-gel” or “nanogel-in-hydrogel” hierarchical architecture that mimics the natural ECM’s multiscale organization (Figure 1) [6].

The molecular weight of HA critically influences its biological functionality, creating a complex duality in its therapeutic applications. Low-molecular-weight HA (LMW-HA, <100 kDa) tends to promote cell proliferation and angiogenesis but may stimulate inflammatory responses, while high-molecular-weight HA (HMW-HA, >1000 kDa) exhibits superior anti-inflammatory and immunosuppressive properties [7]. This molecular-weight-dependent bioactivity must be carefully balanced when designing HA-based nanoencapsulation systems. HA-based formulations have shown remarkable therapeutic outcomes, including drug release sustained up to 7 days and wound closure rates exceeding 90% in animal models [8].

Recent comprehensive reviews reflect the rapid evolution of nanoencapsulation systems. Valachová et al. (2025) detailed hyaluronan (HA)-based nanoencapsulation for smart drug release, emphasizing HA’s versatility in forming nanogels, polymeric micelles, and lipid-polymer hybrid nanoparticles, with a focus on stimuli-responsive release and CD44-mediated targeting [5]. Luo et al. (2026) systematically reviewed the formation mechanisms, controlled release profiles, and applications of HA-based delivery systems, highlighting the importance of molecular weight and chemical modification for optimal therapeutic outcomes [9]. Heera et al. (2026) covered the chemistry, synthesis, and biomedical implementation of HA-based biomaterials, providing fundamental insights into structure–function relationships crucial for designing advanced therapeutic systems [4].

Beyond HA-specific reviews, the broader nanoencapsulation literature provides crucial methodological frameworks. For instance, Hassan et al. (2025) reviewed microencapsulation processes, methods, properties, and applications across various polymeric systems, establishing foundational principles for encapsulation technologies, including matrix-type and vesicular-type systems [10]. Yoon et al. (2025) specifically addressed dual-drug delivery systems utilizing hydrogel-nanoparticle composites, highlighting recent advances in combination therapy approaches and the key applications of these integrated platforms [11]. Furthermore, Parveen et al. (2026) offered a comprehensive overview of nanoparticle–hydrogel composites for precision medicine, emphasizing design principles, therapeutic potential, and the challenges associated with translating these hierarchical systems to clinical practice [12].

These recent reviews highlight the increasing importance of multiscale therapeutic platforms. However, despite abundant information on individual components like nanoencapsulation systems or hydrogel scaffolds, a significant gap remains in comprehensive reviews that specifically address integrating HA-based nanoencapsulation within 3D hydrogel constructs as a unified therapeutic strategy. Most existing reviews treat nanoencapsulation and hydrogel systems separately or focus exclusively on one scale, thus failing to provide a holistic perspective on how these systems can be synergistically combined for hierarchical control over therapeutic delivery.

This review aims to bridge the nano and macro scales of HA-based encapsulation by providing a comprehensive overview of strategies to integrate HA-based nanoencapsulation systems into macroscopic 3D hydrogel constructs. We explore the design of composite systems where nanoparticles serve as reservoirs for controlled, stimuli-responsive release of therapeutics within a supportive hydrogel matrix. The review highlights applications in 3D bioprinting, injectable cell delivery systems, organ-on-a-chip disease models, cancer therapy, and tissue regeneration, culminating in a forward-looking discussion of challenges and opportunities for clinical translation. By systematically addressing the integration of HA-based nanoencapsulation within 3D hydrogel constructs, this review fills a critical gap in the literature and provides a roadmap for researchers developing next-generation multiscale therapeutic platforms.

## 2. Fundamentals of HA-Based Nanoencapsulation

### 2.1. Molecular Properties Enabling Nanoencapsulation

HA’s linear polysaccharide structure—composed of repeating disaccharide units of D-glucuronic acid and N-acetyl-D-glucosamine—can be chemically modified to form amphiphilic copolymers that self-assemble into nanoparticles in aqueous environments. Hydrophobic modification of HA (e.g., with cholesterol, fatty acids, or polylactic acid) enables the formation of self-aggregated HA nanogels through hydrophobic core formation, with the hydrophilic HA shell providing colloidal stability and CD44-targeting capability [5]. These nanogels typically range from 50–300 nm in diameter and can encapsulate hydrophobic drugs, proteins, and nucleic acids within their core while presenting a biocompatible, receptor-interactive surface [13].

**Figure 2:**
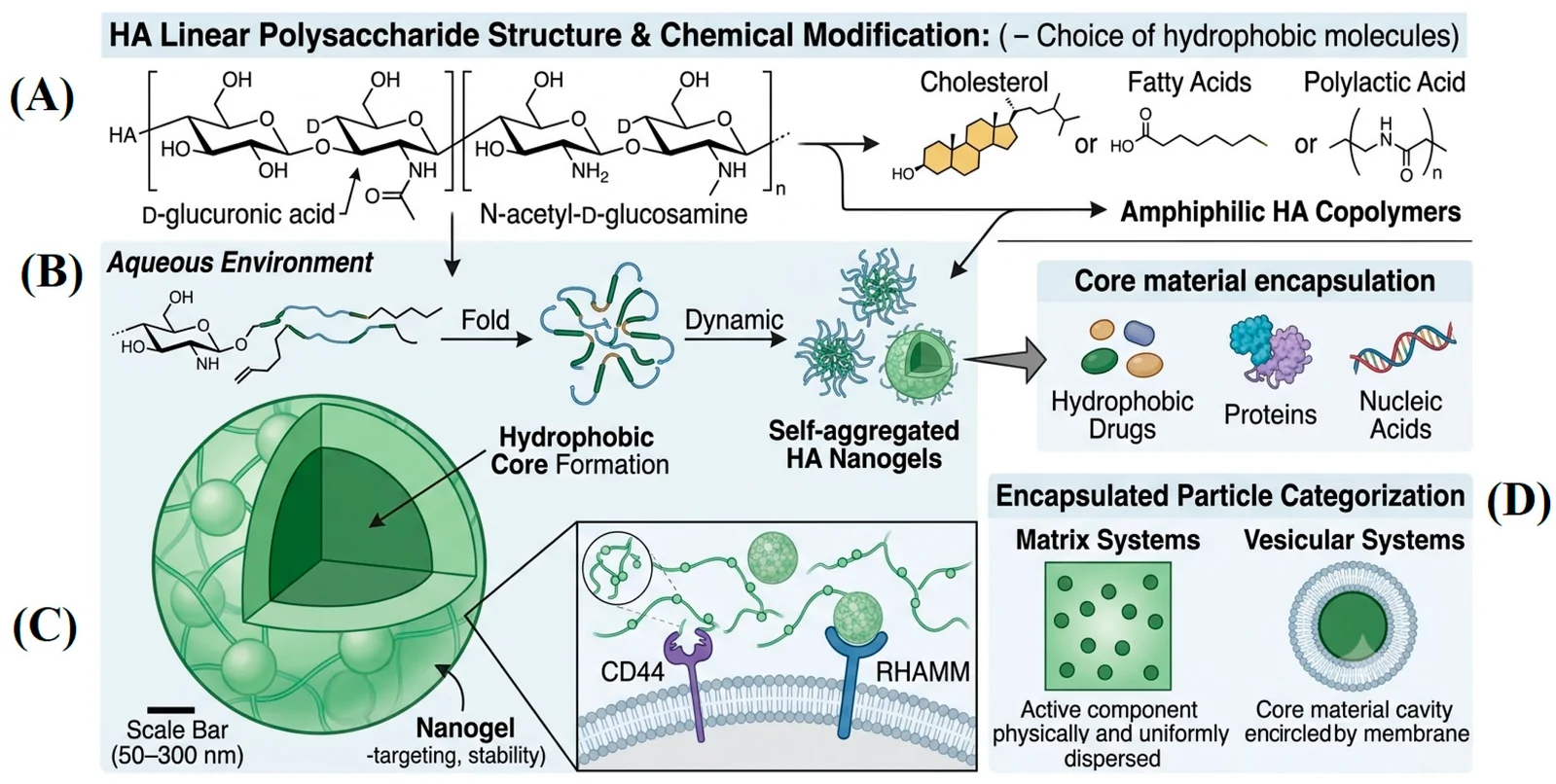
Detailed visualization of hyaluronan chemical modification strategies for nanoparticle and nanogel formation. (**A**) Chemical structure of the native linear HA polysaccharide, composed of repeating disaccharide units of D-glucuronic acid and N-acetyl-D-glucosamine. Hydrophobic modification (e.g., with cholesterol, fatty acids, or polylactic acid) at the hydroxyl or carboxyl groups enables the formation of amphiphilic HA copolymers. (**B**) Mechanism of self-assembly in an aqueous environment, illustrating how amphiphilic copolymers form self-aggregated nanogels with a hydrophobic core for cargo loading and a hydrophilic HA shell for colloidal stability and CD44-targeting. (**C**) Size range and internal structure of a representative nanogel, with a detailed core–shell cross-section. (**D**) Comparative schematic of nanoencapsulation systems: highlighting matrix systems (uniform dispersion of core material) versus vesicular systems (core material cavity encircled by membrane), positioning HA nanogels as versatile matrix systems for encapsulating hydrophobic drugs, proteins, and nucleic acids. Figure concept was developed using Google Gemini (3.7 Flash) for initial schematic suggestions, followed by extensive author editing and refinement. Molecular structures were prepared using ChemDraw 26.0. No AI-generated images were used directly. For AI usage disclosure, see the statement at the end of the manuscript.

HA-based nanoencapsulation systems represent a sophisticated approach to drug delivery that capitalizes on the unique properties of this natural polysaccharide. The encapsulation process involves entrapment of a core material—which may exist in liquid, solid, or gaseous state—within polymeric coatings. Based on the dispersion of the core material, encapsulated particles can be categorized into matrix systems, wherein an active component is physically and uniformly dispersed, and vesicular systems, characterized by the confinement of the core material within a cavity encircled by a polymer membrane [10] (Figure 2).

The advantages of HA-based nanoencapsulation are multifaceted:

- Protection from degradation: Nanostructures protect drugs from hydrolytic degradation that often occurs in aqueous biological environments such as blood plasma and the gastrointestinal tract [5].
- Reduced metabolism: Nanostructures restrain drugs from first-pass metabolism and increase blood residence time [14].
- Enhanced penetration: Their nano-size allows efficient penetration through tissues and biological barriers [15].
- Targeted delivery: Surface modification with HA enables CD44 receptor-mediated targeting [16].

HA-based formulations have demonstrated enhanced bioavailability of encapsulated drugs by 2-3-fold compared with free drugs. The CD44 receptor-targeting capability of HA has been particularly exploited in cancer therapy, where HA-functionalized nanocarriers have shown enhanced tumor accumulation by 5.4–12.7-fold compared with non-targeted systems [8,17].

### 2.2. Chemical Modification Strategies for Nanoencapsulation

The chemical versatility of HA enables diverse modification strategies that enhance its functionality as a nanocarrier. A summary of common HA modification types, functional groups, and their applications is provided in Table 1. Chemical modifications at specific sites using techniques such as deacetylation, amidation, esterification, and oxidation allow for precise tuning of properties [18]. These modifications facilitate the formation of various nanostructures including HA-coated nanoparticles, HA-modified liposomes, HA-based nanogels, and HA-functionalized polymeric carriers [19]. Recent studies have demonstrated the remarkable potential of HA-based nanoencapsulation across diverse therapeutic areas. Xu et al. developed an injectable HA-tyramine hydrogel loaded with interferon α2a, demonstrating three times higher drug concentration in plasma and tumor tissues compared with solution administration, effectively inhibiting tumor growth and angiogenesis [20]. Zhang et al. prepared an HA-fibrin composite hydrogel that stably dispersed DNA carrier complexes, achieving high transfection efficiency both in vivo and in vitro [21]. HA-modified cationic polymers have been shown to effectively target tumors, shield positive charges, improve transfection efficiency, and reduce toxicity [22].

The loading capacity and encapsulation efficiency of HA-based nanoencapsulation systems are critically dependent on multiple factors, including HA molecular weight, degree of hydrophobic modification, drug–polymer compatibility, and formulation conditions [37]. Encapsulating hydrophilic agents (e.g., proteins, nucleic acids), however, typically results in lower loading capacities and encapsulation efficiencies compared to hydrophobic drugs. This necessitates the development of specialized strategies such as complex coacervation, electrostatic assembly, or double emulsion techniques [38,39,40].

### 2.3. Molecular Weight-Dependent Bioactivity

The molecular weight of HA significantly influences both nanoencapsulation efficiency and biological activity. Understanding this relationship is crucial for designing optimal therapeutic systems, as summarized in Table 2. Low-molecular-weight HA (LMW-HA, <100 kDa) promotes inflammation, immunostimulation, and angiogenesis, making it suitable for wound healing and cancer sensitization applications. In contrast, high-molecular-weight HA (HMW-HA, >1000 kDa) exhibits anti-inflammatory and immunosuppressive properties, rendering it ideal for tissue protection in conditions such as osteoarthritis and inflammatory bowel disease. Medium-molecular-weight HA (100–1000 kDa) displays balanced bioactivity and is commonly employed for general drug delivery applications. LMW-HA fragments have shown the ability to sensitize cancer cells to chemotherapeutic drugs, presenting opportunities for combination therapy approaches [41]. Conversely, HMW-HA exhibits superior anti-inflammatory properties, making it ideal for protecting tissues from excessive inflammation in conditions such as osteoarthritis and inflammatory bowel disease [42]. The differential biological effects of HA are largely mediated through its interactions with cell surface receptors, particularly CD44 and RHAMM, which exhibit varying affinities depending on HA molecular weight.

The molecular weight (MW) of hyaluronic acid (HA) critically determines its biological function and therapeutic efficacy due to MW-dependent bioactivity. This mediates differential binding affinities to cell surface receptors like CD44 and RHAMM, and influences Toll-like receptor 4 (TLR4) signaling pathways [43].

The binding affinity of HA to CD44 is highly dependent on molecular weight. Studies have demonstrated that high-molecular-weight HA (HMW-HA, >1000 kDa) exhibits higher multivalent binding avidity to CD44 compared to low-molecular-weight HA (LMW-HA, <100 kDa). Specifically, the dissociation constants (Kd) for HA-CD44 interactions range from approximately 10^−6^ to 10^−8^ M, with HMW-HA showing Kd values in the low nanomolar range (Kd ≈ 2–10 nM) while LMW-HA fragments exhibit Kd values in the micromolar range (Kd ≈ 1–50 µM). This difference in binding affinity translates to significant differences in downstream signaling and biological responses [44].

The half-maximal effective concentration (EC_50_) for HA-induced CD44 clustering and signaling varies with molecular weight. For HMW-HA, the EC_50_ for CD44-mediated cell adhesion is approximately 0.5–2 µg/mL, while for LMW-HA fragments (10–50 kDa), the EC_50_ is approximately 10–50 µg/mL [45]. Similarly, RHAMM binding to HA shows molecular weight dependence, with HMW-HA exhibiting higher binding affinity (Kd ≈ 50–100 nM) compared to LMW-HA (Kd ≈ 500–1000 nM) [45,46].

The differential biological effects of HA are mediated through downstream signaling pathways. HMW-HA engagement of CD44 promotes anti-inflammatory cytokine production, with studies reporting 2-5-fold increases in IL-10 and TGF-β1 secretion from macrophages in response to HMW-HA (1000–2000 kDa) compared to untreated controls [47]. In contrast, LMW-HA fragments (10–50 kDa) stimulate pro-inflammatory cytokine production, with TNF-α and IL-6 levels increasing by 3-8 fold and IL-12 levels increasing by 2-4-fold compared to untreated controls [48,49].

The molecular-weight-dependent effects on cell proliferation and migration are equally significant. LMW-HA (10–100 kDa) stimulates endothelial cell proliferation and tube formation by 2-4 fold compared to HMW-HA, while HMW-HA (>1000 kDa) inhibits endothelial cell proliferation by 40–60% [43]. In cancer cells, LMW-HA fragments (20–100 kDa) have been shown to increase cell migration and invasion by 3-5 fold through CD44 and RHAMM activation, while HMW-HA (>2000 kDa) reduces cancer cell migration by 50–70% [50].

Toll-like receptor 4 (TLR4) mediates inflammatory responses to LMW-HA fragments. The EC_50_ for TLR4 activation by LMW-HA (20–50 kDa) is approximately 50–100 µg/mL, with maximal NF-κB activation occurring at 200–500 µg/mL [51]. This TLR4-dependent signaling leads to the production of pro-inflammatory cytokines, with TNF-α levels increasing by 5-10 fold and IL-1β levels increasing by 3-8 fold. In contrast, HMW-HA (>1000 kDa) does not activate TLR4 and can actually inhibit LPS-induced TLR4 signaling by 40–70%, providing a mechanistic basis for its anti-inflammatory properties [52].

**Table 2:** Molecular Weight-Dependent Biological Effects and Therapeutic Implications of Hyaluronic Acid (HA).

Molecular Weight Range	Biological Effects	Therapeutic Implications	Ref.
LMW-HA (<100 kDa)	Promotes inflammation, immunostimulation, angiogenesis	Wound healing, cancer sensitization, immunotherapy	[53]
Medium MW HA (100–1000 kDa)	Balanced bioactivity	General drug delivery, tissue engineering scaffolds	[54]
HMW-HA (>1000 kDa)	Anti-inflammatory, immunosuppressive	Tissue protection, osteoarthritis, inflammatory bowel disease	[55]

## 3. Design and Synthesis of 3D HA Hydrogels

### 3.1. Hydrogel Formation and Crosslinking Strategies

Hydrogels are cross-linked polymer networks that form soft, highly porous 3D structures with key properties including swelling, wettability, mechanical stiffness, biocompatibility, biodegradability, and biological functionalities such as cell adherence, migration, proliferation, differentiation, and angiogenesis [56]. These characteristics make hydrogels ideal for mimicking the extracellular matrix in tissue engineering and biological research [57]. HA hydrogels are fabricated through diverse crosslinking strategies that can be broadly categorized into covalent (chemical) and non-covalent (physical) approaches. Covalent crosslinking includes divinyl sulfone (ether bond formation), carbodiimide-mediated coupling (amide bonds), methacrylation (photocrosslinking), and Schiff base reactions (reversible imine bonds), while enzymatic crosslinking via HRP-catalyzed tyramine coupling enables rapid in situ gelation [58,59,60,61]. Non-covalent crosslinking mechanisms are equally important, particularly for nanoencapsulation systems, and include hydrogen bonding that underpins HA’s viscoelastic properties, hydrophobic interactions driving self-assembly of amphiphilic HA derivatives into nanogels and micelles, ionic coordination with multivalent ions, host–guest interactions such as cyclodextrin-adamantane systems for dynamic networks, and electrostatic interactions forming polyelectrolyte complexes with cationic polymers [62,63,64,65,66,67,68,69,70]. These non-covalent interactions govern the formation of matrix-type and vesicular-type nanoencapsulation systems and enable stimuli-responsive release mechanisms, self-healing properties, and shear-thinning behavior essential for injectable applications. Both covalent and non-covalent crosslinking methods yield 3D networks with water content exceeding 96%, high porosity, and tunable mechanical properties ranging from 1–100 kPa to match soft tissues or more rigid constructs [71]. Critically, the choice of crosslinking chemistry directly influences the integration strategy for nanoencapsulation systems, as it determines network mesh size, degradation kinetics, and available functional groups for nanoparticle incorporation or covalent attachment.

### 3.2. Mechanical and Structural Properties

Methacrylated HA (MeHA) crosslinked via UV exposure enables precise control over mechanical properties, with stiffness tunable by varying the degree of methacrylation (typically 10–60%) and polymer concentration, allowing stiffness modulation from ~1 kPa to over 100 kPa to match various tissue types [72]. Thiol-modified HA hydrogels can mimic the physiological stiffness of healthy liver ECM (~1–5 kPa), enhancing cellular adhesion and proliferation, especially when combined with liver ECM components [73]. Adipic dihydrazide-HA hydrogels have been developed as chemoattractant and anticancer drug-loaded systems to attract and eradicate infiltrative glioma cells, demonstrating the versatility of HA-based hydrogels for targeted therapeutic applications [74].

The incorporation of HA into composite hydrogel systems has enabled the creation of biomimetic environments with tunable structural and biological properties, as summarized in Table 3. Alginate-HA hydrogels have demonstrated HA molecular-weight-dependent invasion behavior of breast cancer cells, with 117 kDa HA hydrogels inhibiting cell migration and invasion while 35 kDa HA exhibited upregulation of epithelial–mesenchymal transition markers, highlighting the critical role of HA molecular weight in modulating cellular behavior [54]. Gelatin-methacrylamide-HA hydrogels mimic glioblastoma ECM, with tunable mechanical properties achieved by altering crosslinking density, providing a physiologically relevant platform for studying cancer cell invasion and drug response [75]. HA-chitosan injectable composite hydrogels, formed via Schiff-base reaction, have shown excellent self-healing properties and good cytocompatibility supporting cell adhesion and proliferation for bone repair tissue engineering [76]. HA-collagen composite hydrogels provide ECM-mimetic environments for skin and wound healing applications, leveraging the natural synergy between these two ECM components.

**Figure 3:**
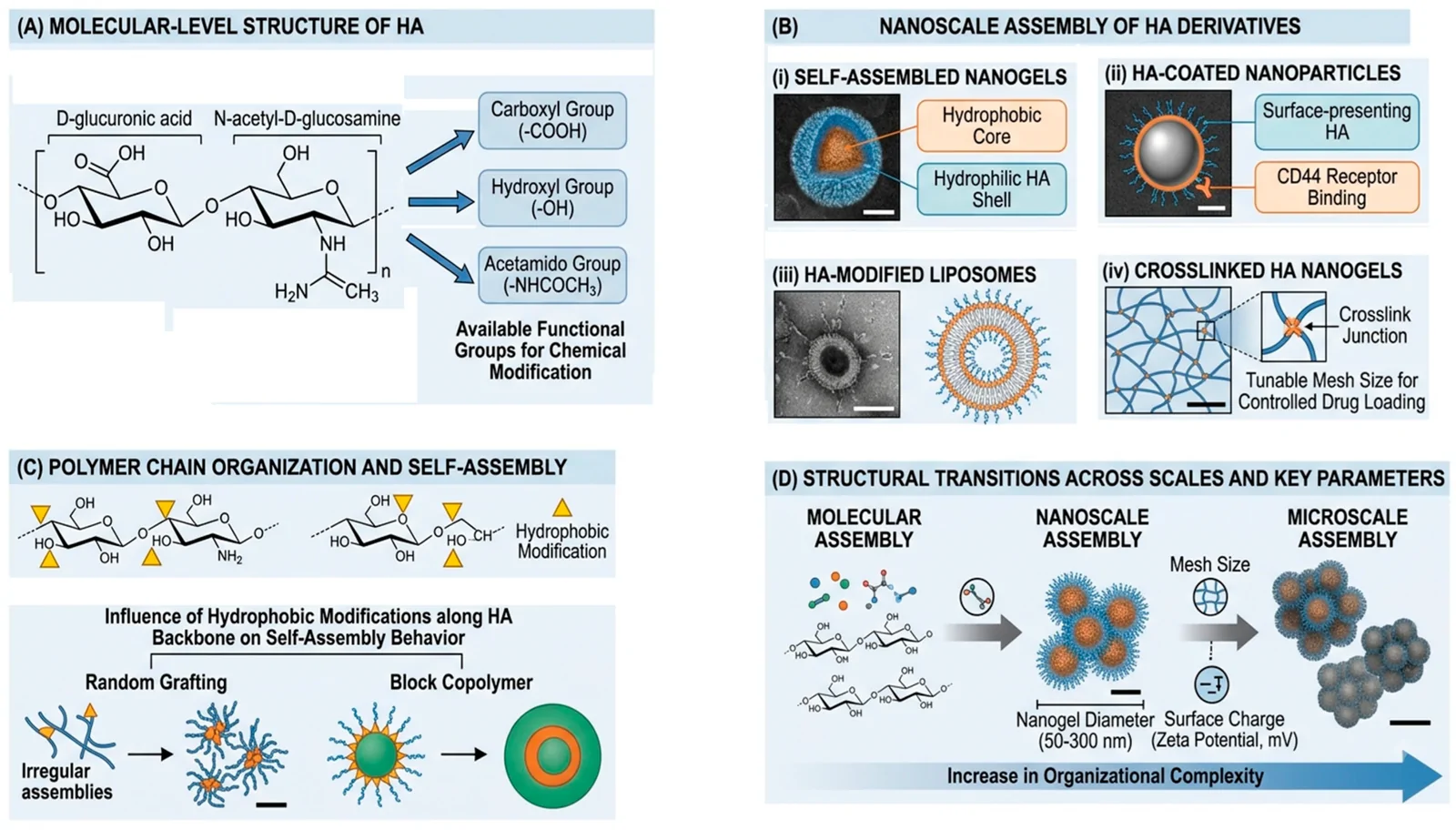
Schematic representation of the hierarchical structural characteristics of HA-based nanomaterials. (**A**) Molecular-level structure showing the repeating disaccharide units of HA (D-glucuronic acid and N-acetyl-D-glucosamine) with functional groups available for chemical modification (hydroxyl, carboxyl, and acetamido groups). (**B**) Nanoscale assembly illustrating the formation of various nanostructures from HA derivatives: (i) self-assembled nanogels with hydrophobic core and hydrophilic HA shell, (ii) HA-coated nanoparticles with surface-presenting HA for CD44 targeting, (iii) HA-modified liposomes for enhanced circulation and targeting, and (iv) crosslinked HA nanogels with tunable mesh size for controlled drug loading. (**C**) Polymer chain organization showing the distribution of hydrophobic modifications along the HA backbone and their influence on self-assembly behavior. (**D**) Structural transitions from molecular to nanoscale to microscale assemblies, demonstrating the hierarchical organization of HA-based materials. Key structural parameters including nanogel diameter (50–300 nm), mesh size, and surface charge (zeta potential) are indicated. Figure concept was developed using Google Gemini (3.7 Flash) for initial schematic suggestions, followed by extensive author editing and refinement based on application examples from the literature. No AI-generated images were used directly. For AI usage disclosure, see the statement at the end of the manuscript.

The biological performance of these composite systems is further modulated by HA’s interactions with cell surface receptors (Figure 3). For instance, CD44 engagement promotes cell adhesion, migration, and proliferation, while RHAMM activation influences cell motility, tissue organization, and matrix remodeling. Additionally, integrin receptors (e.g., αvβ3, α5β1) mediate cell–ECM interactions in HA-collagen and HA-gelatin composites, facilitating cell adhesion and mechanotransduction, while TLR4 activation by LMW-HA fragments can modulate inflammatory responses in wound healing and cancer models, creating a complex regulatory network that must be carefully considered in system design.

### 3.3. HA-Based Bioinks for 3D Bioprinting

Recent advances have demonstrated the potential of HA-based hydrogels for advanced therapeutic applications through 3D bioprinting. The development of HA-based bioinks has opened new avenues for tissue engineering, with methacrylated collagen and thiolated-HA bioinks enhancing cell proliferation and enabling precise spatial control over cell distribution [81]. Tyramine-derivative HA bioinks integrated with aligned collagen fibers have been shown to facilitate the differentiation of mesenchymal stem cells into chondrocytes [73].

HA-based hydrogels provide a biomimetic environment that supports cell growth and allows for the diffusion of nutrients and waste. The methacrylation of HA enables photo-crosslinking during printing, allowing for precise spatial control and the creation of complex architectures. HA-based bioinks have been developed with varying molecular weights to achieve desired mechanical properties and biological responses, with low-molecular-weight HA hydrogels showing higher glioblastoma invasion while high-molecular-weight HA hydrogels exhibiting different biological behaviors [82].

While HA-based bioinks have demonstrated remarkable potential for 3D bioprinting, synthetic polymer materials offer complementary advantages that should not be overlooked. Synthetic polymers provide precise tunability through controlled molecular weight, composition, and functionality, enabling predictable and reproducible printing behavior [83]; mechanical robustness through materials such as polycaprolactone (PCL), PLGA, and PEG that supply structural strength often lacking in pure HA hydrogels [84]; thermal responsiveness enabling processing through melt-based techniques like fused deposition modeling (FDM) that are not applicable to hydrogels [85]; and long-term stability providing sustained structural support over extended periods, complementing HA’s biodegradable nature. These advantages make synthetic polymers valuable components in hybrid HA-synthetic bioink systems, enabling the fabrication of constructs with optimized mechanical, degradation, and biological properties for applications ranging from load-bearing tissue engineering to drug delivery and disease modeling [86].

#### Hybrid HA-Synthetic Polymer Systems for 3D Printing

Recent advances have focused on combining HA with synthetic polymers to create hybrid bioinks that leverage the advantages of both material classes. HA-PEG hydrogels offer improved printability and mechanical properties while maintaining CD44-targeting capability, with PEG segments providing steric stabilization and reducing non-specific protein adsorption [87]. HA-PLGA composites incorporate PLGA nanoparticles loaded with therapeutic agents for controlled drug release kinetics while the HA matrix supports cell growth and tissue integration [88]. HA-PCL scaffolds combine HA hydrogels printed onto electrospun PCL nanofibers, merging the mechanical strength of PCL with the biological activity of HA in defined macro- and microstructures [89]. HA-Pluronic blends leverage the thermoresponsive properties of Pluronic F127 to enable temperature-dependent gelation and improved printability when blended with HA methacrylate [90]. HA-Polyurethane systems utilize multi-layer scaffolds combining polyurethane nanofibers with HA-gelatin hydrogels for cardiovascular tissue engineering, where polyurethane provides mechanical support and elasticity while HA-gelatin enhances biocompatibility and endothelial cell adhesion [91]. These hybrid systems demonstrate that integrating synthetic polymers with HA enables fabrication of constructs with tailored mechanical, degradation, and biological properties optimized for diverse applications, ranging from load-bearing tissue engineering to drug delivery and disease modeling, significantly expanding the capabilities of 3D printing beyond purely biological materials [92].

## 4. Strategies for Integrating Nano- and Macroscale Systems

**Figure 4:**
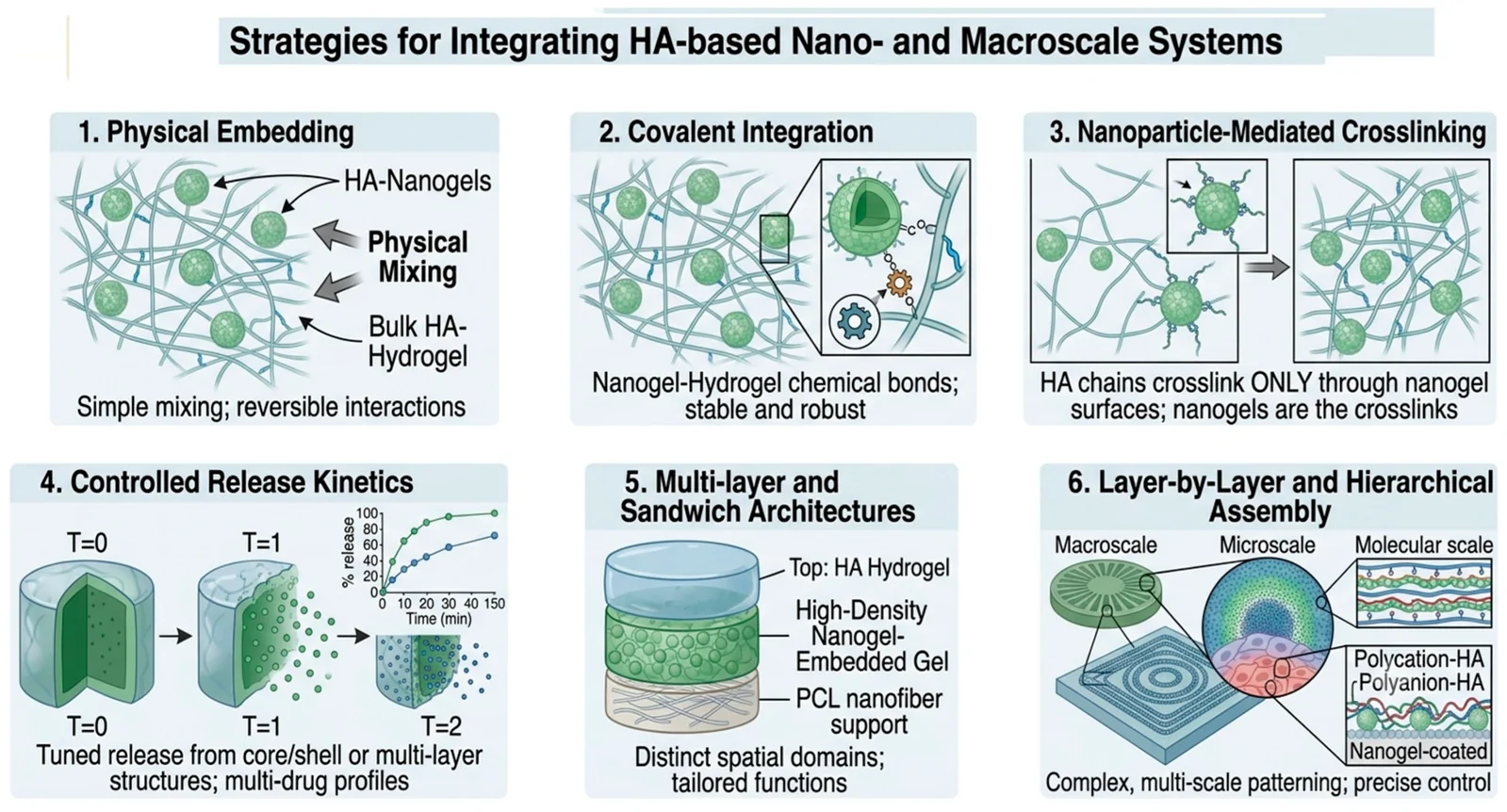
Comprehensive overview of technical strategies for integrating HA-based nanogels into 3D macroscopic hydrogel constructs, enabling synergistic material properties. (**1**) Simple physical embedding via physical mixing; characterized by reversible interactions. (**2**) Covalent integration, where nanogels are chemically bonded within the hydrogel network, offering stability and robustness. (**3**) Nanoparticle-mediated crosslinking, in which HA polymer chains are crosslinked only through interactions at the nanogel surfaces, with the nanogels acting as the central crosslinks. (**4**) Tuning release kinetics from complex composite systems, demonstrating independent, controlled release profiles (e.g., pH-triggered or multi-drug release) from specific matrix domains and time-dependent degradation (T = 0, T = 1, T = 2). (**5**) Advanced fabrication strategies, including multi-layer and sandwich architectures with distinct spatial domains for tailored functions. (**6**) Complex layer-by-layer and hierarchical assembly using microfluidic patterning and sequential polymer deposition to achieve complex, multi-scale, micro-engineered structures. Figure concept was developed using Google Gemini (3.7 Flash) for initial schematic suggestions, followed by extensive author editing and refinement based on strategies reviewed in the literature. No AI-generated images were used directly. For AI usage disclosure, see the statement at the end of the manuscript.

The integration of HA-based nanoencapsulation systems into 3D hydrogel constructs represents a frontier in biomaterials engineering, offering synergistic benefits that transcend the capabilities of either system alone. Several strategies have been developed to achieve this integration, each with distinct advantages and considerations (Figure 4) [93].

### 4.1. Physical Embedding of HA-Nanoparticles Within HA-Hydrogels

The most straightforward approach involves dispersing pre-formed HA-based nanoparticles within an HA hydrogel precursor solution prior to gelation. This method preserves the independent functionality of both components and allows for modular design [11,94]. For example, spermidine-modified mesoporous polydopamine nanoparticles have been embedded within hyaluronic acid methacrylate/Pluronic F127 double-network hydrogels for periodontitis treatment, combining photothermal antibacterial activity, ROS scavenging, and anti-inflammatory effects in a single injectable platform [95,96]. The nanoparticles remain physically entrapped within the hydrogel mesh, with their release governed by hydrogel degradation and diffusion kinetics.

Similarly, ZnO nanoparticles have been incorporated into o-nitrobenzene-modified HA hydrogels (ZnO@HN) for infected diabetic wound healing. The ~50 nm ZnO particles diffuse from the hydrogel network to scavenge bacteria effectively, while the HA hydrogel provides moist wound healing environment and tissue adhesion [97,98]. The physical embedding approach is versatile but requires careful optimization of nanoparticle concentration to avoid compromising hydrogel mechanical properties or injectability.

Recent studies have explored the incorporation of nanoparticles into HA-based hydrogels to modulate material properties. The addition of carbon nanotubes and silica nanoparticles to HA hydrogels has been shown to decrease swelling capability while increasing Young’s modulus, providing tunable mechanical properties [99]. The presence of nanometric phases within the hydrogel network not only improved mechanical properties but also enhanced cell growth in early culture stages, suggesting potential benefits for tissue engineering applications. These nanohybrid scaffolds demonstrated promising results for neural tissue regeneration, highlighting the versatility of nanoparticle–hydrogel composites [100].

### 4.2. Covalent Integration of Nanogels Within Hydrogel Networks

A more sophisticated strategy involves covalently tethering HA-nanogels within the 3D hydrogel matrix, preventing uncontrolled release and enabling localized, sustained drug delivery [101]. Methacrylated HA nanogels embedded within gelatin methacrylate (GelMA) matrices via extrusion-based 3D printing followed by UV crosslinking represent a prime example. In this hierarchical system, nanogels remain covalently bound within the matrix, reducing burst release from ~62% (hydrogel alone) to ~37% over 7 days while maintaining structural integrity and printability [102].

Covalent integration is particularly valuable for applications requiring long-term drug delivery, such as chronic disease management or tissue engineering scaffolds where sustained growth factor presentation is critical. The methacrylation degree of both the nanogel and hydrogel components must be precisely controlled to achieve optimal crosslinking density without compromising biocompatibility [103].

HA’s high density of hydroxyl and carboxyl functional groups facilitates extensive inter- and intramolecular hydrogen bonding, forming a three-dimensional network that is responsible for its viscoelasticity and moisture-retentive properties. These functional groups can be exploited for chemical conjugation of nanoparticles, enabling the creation of composite systems with enhanced stability and controlled release properties [104]. Hydrogel nanoparticles crosslinked with hydrophilic polymeric networks, known as nanogels, have high encapsulation capacity and swelling properties, making them potential drug delivery materials [105].

The integration of nanoparticles into HA hydrogels enables sophisticated control over drug release kinetics. This control is achieved through multiple mechanisms working in concert: the hydrogel network provides a diffusion barrier, nanoparticle encapsulation protects the drug and modulates its release, and the degradation of both components contributes to the overall release profile [106]. Xu et al. developed an injectable HA-tyramine hydrogel loaded with interferon α2a (IFN-α2a) for liver cancer therapy, demonstrating controlled release over 7 days with only 20–25% burst release in the first 24 h, compared to >80% release from solution administration. The hydrogel system maintained therapeutic drug concentrations in plasma and tumor tissues for up to 7 days, achieving three times higher drug concentration compared with solution administration, effectively inhibiting tumor growth and angiogenesis [20]. Shakib et al. developed a multi-layered scaffold combining electrospun polyurethane nanofibers with HA-gelatin hydrogels, achieving controlled release of methylprednisolone over 14 days with 80% cumulative release. The release profile followed near-zero-order kinetics (R^2^ = 0.98) from day 2 to day 14, with a minimal burst release of 15% in the first 24 h, demonstrating the potential for sustained therapeutic delivery in cardiovascular applications [91]. Zhang et al. prepared HA-fibrin composite hydrogels that stably dispersed DNA carrier complexes, achieving high transfection efficiency both in vivo and in vitro. The system demonstrated enhanced release in acidic conditions (pH 5.5) compared to physiological pH (7.4), with the release rate at pH 5.5 being 2.5× higher than at pH 7.4, showing 70% cumulative release at pH 5.5 versus 25% at pH 7.4 over 7 days. Sun et al. developed pH-responsive self-assembled nanoparticles for tumor-targeted drug delivery, demonstrating 3.5 × higher releases in the presence of hyaluronidase [21].

The release kinetics showed that degradation of the HA matrix by hyaluronidase enzyme (100 U/mL) increased doxorubicin release from 30% to 85% over 48 h, highlighting the potential for enzyme-triggered drug delivery in the tumor microenvironment [107]. A more sophisticated strategy involves covalently tethering HA-nanogels within the 3D hydrogel matrix, preventing uncontrolled release and enabling localized, sustained drug delivery [105]. Methacrylated HA nanogels embedded within gelatin methacrylate (GelMA) matrices via extrusion-based 3D printing followed by UV crosslinking represent a prime example [108]. In this hierarchical system, nanogels remain covalently bound within the matrix, reducing burst release from ~62% (hydrogel alone) to ~37% over 7 days while maintaining structural integrity and printability [6]. Covalent integration is particularly valuable for applications requiring long-term drug delivery, such as chronic disease management or tissue engineering scaffolds where sustained growth factor presentation is critical. The methacrylation degree of both the nanogel and hydrogel components must be precisely controlled to achieve optimal crosslinking density without compromising biocompatibility [103].

### 4.3. Nanoparticle-Mediated Crosslinking

An innovative approach utilizes nanoparticles themselves as crosslinking agents within the hydrogel network. Gold nanoparticles (AuNPs), for instance, can crosslink thiol-modified HA through multivalent interactions, creating hybrid hydrogels with tunable mechanical properties [109]. This strategy not only integrates the nanoscale and macroscale components but also introduces additional functionalities such as photothermal responsiveness or imaging contrast.

In a notable application, Au-triptolide nanoparticles were integrated into HA hydrogels for intraarticular treatment of rheumatoid arthritis. The AuNPs served dual roles: as crosslinking agents contributing to hydrogel stability and as photothermal transducers enabling NIR-triggered drug release [110]. This “theranostic” platform combined anti-inflammatory, chondroprotective, and imaging capabilities, demonstrating the synergistic potential of nanoparticle–hydrogel integration.

### 4.4. Controlled Release Kinetics from Composite Systems

The integration of nanoparticles into HA hydrogels enables sophisticated control over drug release kinetics. This control is achieved through multiple mechanisms working in concert: the hydrogel network provides a diffusion barrier, nanoparticle encapsulation protects the drug and modulates its release, and the degradation of both components contributes to the overall release profile [12].

HA-based formulations have demonstrated drug release sustained up to 7 days, representing a significant improvement over conventional delivery systems. Controlled and responsive release mechanisms can be engineered to respond to physiological changes in the human body, such as variations in pH, enzyme activity, and temperature [111]. This engineering provides control over the rate at which the active ingredient is released and absorbed, thereby maximizing therapeutic efficacy and minimizing adverse effects. The release of the core material from nano/microcapsules can be engineered to respond to specific biological triggers, enabling smart drug delivery systems that deliver drug molecules selectively to the desired site while reducing dosage repetition and minimizing side effects [112].

HA-based stimulus-responsive systems have been extensively developed, particularly pH-responsive systems that exploit the acidic tumor microenvironment for targeted drug release [113]. ROS-responsive HA-bilirubin nanoparticles have enabled tumor-specific release of doxorubicin, exhibiting synergistic anticancer effects [114]. These sophisticated designs showcase a refined “superselective targeting-microenvironmental triggering” paradigm with significant translational potential.

### 4.5. Multi-Layer and Sandwich Architectures

Advanced strategies have employed multi-layer and sandwich architectures to achieve synergistic functionality. These designs combine the mechanical support of electrospun fibers with the biological activity of hydrogels and the targeted delivery of nanoparticles. Shakib et al. developed a multi-layered scaffold combining electrospun polyurethane nanofibers containing methylprednisolone with HA-gelatin hydrogels at varying ratios. This 3D structure enhanced porosity and mechanical properties, with increasing the HA:gelatin ratio from 1:10 to 1:2 expanding pore size from ~208 µm to ~489 µm, creating a more favorable microenvironment for cell activities. The elongation at break increased from ~103% in polyurethane alone to ~196% in the composite, showing improvement in viscoelastic behavior. Controlled release of methylprednisolone was demonstrated over 80% delivery within 14 days, highlighting the potential for sustained therapeutic delivery [115].

Biological evaluations with human umbilical vein endothelial cells highlighted the scaffold’s potential to support cell activities, including extensive filopodia anchoring, confirming the biocompatibility of these multi-layer scaffolds. The fiber/hydrogel scaffolding approach not only enhances biocompatibility but also creates a 3D structure that augments bioactivity, contributing to improved overall performance for cardiovascular tissue engineering applications [116].

### 4.6. Layer-by-Layer and Hierarchical Assembly

For applications requiring spatially organized delivery, layer-by-layer (LbL) assembly of nanoparticles within hydrogels offers precise control over construct architecture. This approach enables the creation of gradient or compartmentalized systems where different therapeutic agents are released in a spatiotemporally controlled manner [117]. While less commonly reported for pure HA systems, LbL strategies have been employed with HA-chitosan polyelectrolyte complexes and are readily adaptable to HA-based nanoencapsulation platforms [118].

## 5. Characterization Techniques

The characterization of integrated nano- and macroscale HA-based systems requires sophisticated techniques to evaluate structural, mechanical, and biological properties [119].

### 5.1. Structural Characterization

Fourier transform infrared spectroscopy (FTIR) is commonly used to confirm the chemical structure of HA-based materials and the successful incorporation of nanoparticles. FTIR spectra for HA-based scaffolds with different nanoparticle compositions reveal characteristic peaks corresponding to HA, with slight differences observed due to the presence of nanometric phases [120]. The C-O and asymmetric C=O bonds stretching at 1403 and 1600 cm^−1^, respectively, are typical peaks of HA that can be clearly distinguished in composite materials [121]. Quantitative analysis includes calculation of the degree of substitution (DS) for modified HA derivatives, typically ranging from 5–50% depending on the modification strategy.

Scanning electron microscopy (SEM) and transmission electron microscopy (TEM) are essential for visualizing the morphology and distribution of nanoparticles within the hydrogel matrix. These techniques reveal the porous structure of hydrogels and the integration of nanoparticles, providing information about pore size, nanoparticle distribution, and the overall architecture of the composite system [122]. Quantitative image analysis can determine porosity (>90%), pore size distribution, and nanoparticle density.

Dynamic light scattering (DLS) and zeta potential measurements are crucial for characterizing nanoparticle size distribution and surface charge, which influence colloidal stability and cellular interactions [123].

Nuclear Magnetic Resonance (NMR) Spectroscopy provides detailed structural information, including the degree of chemical modification. For example, the methacrylation degree of HAMA can be quantified by ^1^H NMR, typically ranging from 10–60%, with the degree of substitution directly correlating with crosslinking density and hydrogel mechanical properties [124].

Thermogravimetric Analysis (TGA) provides quantitative information on the thermal stability and composition of HA-based nanocomposite systems. Typical TGA profiles show 3–5% weight loss below 100 °C (water), 10–30% weight loss between 200–400 °C (polymer degradation), and residual mass corresponding to inorganic nanoparticle content [125]. The degradation temperature (Td) typically ranges from 250–350 °C for HA derivatives, with nanoparticle incorporation often increasing Td by 20–50 °C due to improved thermal stability [126]. Differential Scanning Calorimetry (DSC) provides information on the thermal transitions of HA-based systems, including glass transition temperature (Tg), melting temperature (Tm), and crystallization behavior. The Tg of HA typically ranges from 60–100 °C depending on molecular weight and water content, with chemical modifications affecting thermal properties [127].

X-ray Diffraction (XRD) is used to assess the crystallinity of HA-based materials and the incorporation of crystalline nanoparticles. HA typically shows a broad amorphous halo with limited crystallinity (5–15%), while crystalline nanoparticles (e.g., Au, ZnO, BaTiO_3_) show characteristic diffraction peaks [128].

### 5.2. Mechanical Properties

Mechanical characterization is critical for evaluating the performance of HA-based composite systems. Compressive elastic moduli are typically measured to assess the stiffness of hydrogels and the effect of nanoparticle incorporation. The presence of nanometric phases in HA scaffolds has been shown to change the profile of Young’s moduli, with the effect being dependent on nanoparticle concentration and size [129]. Materials with nanometric phases exhibit higher Young’s moduli, which may result in greater biological viability of seeded cells due to increased stiffness that affects cell adhesion and offers cells greater protection from external mechanical stresses [130].

Rheological characterization provides information about the viscoelastic properties of hydrogels, which are critical for injectable and bioprinting applications. HA-based hydrogels exhibit shear-thinning properties that facilitate injection, with the viscoelasticity making them ideal for biological applications such as tissue protection and lubrication of biological interfaces [131].

### 5.3. Swelling and Degradation Studies

Swelling behavior and degradation kinetics are essential parameters that influence drug release and biological performance. Equilibrium water content (EWC) measurements indicate the water uptake capacity of hydrogels, which affects drug release and nutrient diffusion. The addition of nanoparticles to HA hydrogels has been shown to modulate swelling behavior, with the presence of nanoparticles decreasing swelling in liquid DPBS while increasing water uptake from a humid vapor phase [132]. This property can help achieve more controlled water intake than HA alone, resulting in better overall performance as the material is less deformed upon environmental water intake [133].

Degradation studies assess the stability of HA-based composite systems and the release of encapsulated therapeutics. HA is susceptible to enzymatic degradation, particularly by hyaluronidase, which can be exploited for controlled release. The degradation rate can be tuned through chemical modification and crosslinking density, enabling the design of systems with appropriate degradation kinetics for specific applications [134].

### 5.4. Biological Characterization

Biological evaluation of HA-based composite systems is essential for confirming biocompatibility and therapeutic efficacy. Cell viability assays, such as MTS or MTT assays, assess the metabolic activity of cells cultured on or within the hydrogel scaffolds. The presence of nanoparticles in HA hydrogels has been shown not to be detrimental to cell viability and can potentially improve early cell survival, which is critical for the therapeutic success of tissue engineering applications [135].

Cell adhesion, proliferation, and differentiation assays evaluate the biological functionality of HA-based composite systems. The biomimetic properties of HA support cell adhesion and proliferation, with HA-based hydrogels promoting cell adhesion and supporting tissue-specific functions such as chondrogenesis, osteogenesis, neurogenesis, cardiogenesis, and angiogenesis [136]. The incorporation of HA modulates inflammation by binding monocytes and influencing the immune response, further enhancing its potential for regenerative medicine applications [137].

## 6. Advanced Therapeutic Applications

**Figure 5:**
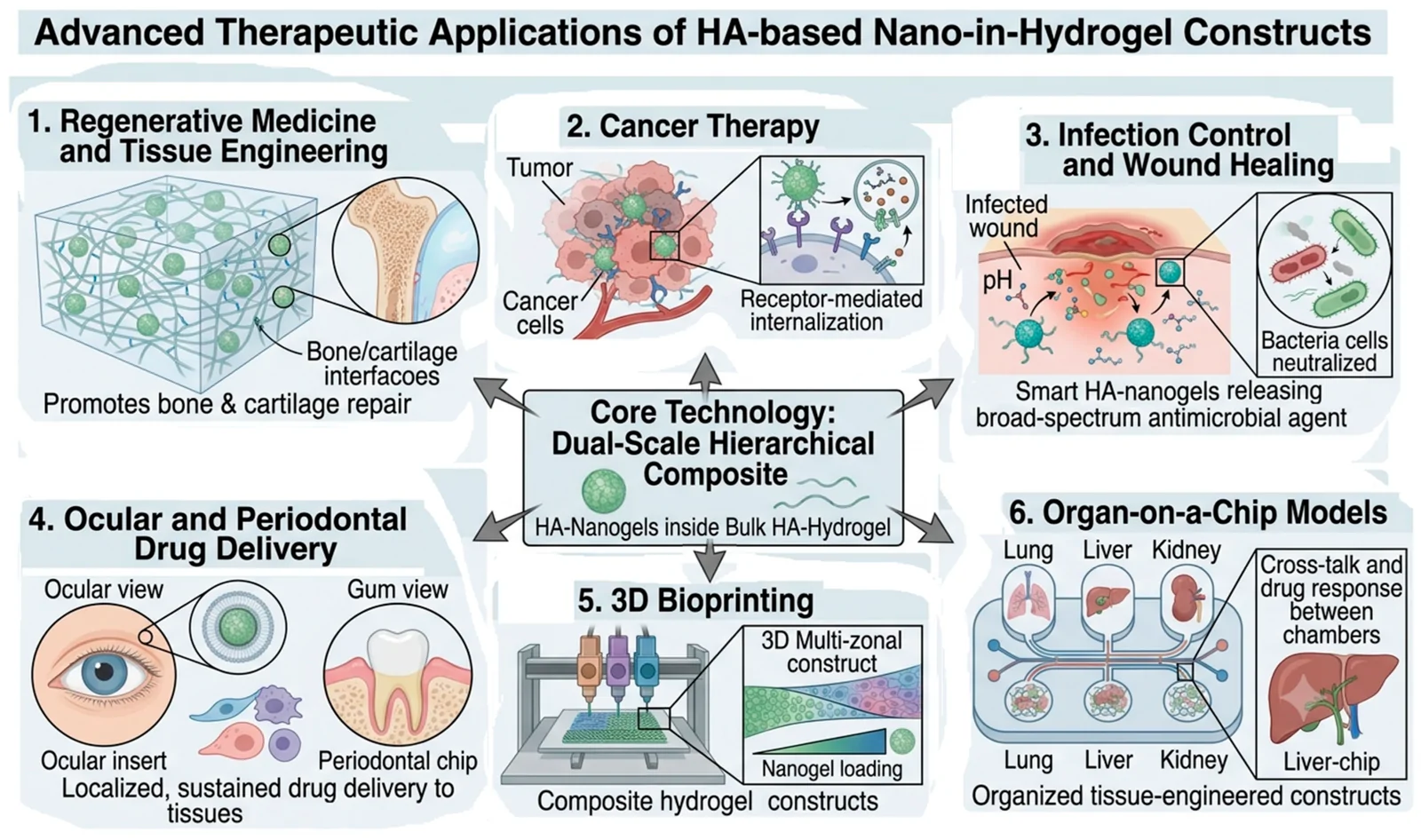
Summary of advanced therapeutic applications and biomedical domains enabled by integrated HA-based nano-in-hydrogel constructs. The diagram illustrates how unique properties, such as native ECM mimicry, receptor targeting (CD44), tunable degradation, and multi-scale drug loading, drive translation across six key areas: (**1**) Regenerative Medicine and Tissue Engineering: multi-zonal scaffolds for bone/cartilage repair; (**2**) Cancer Therapy: CD44 receptor-mediated targeting and core–shell drug delivery; (**3**) Infection Control and Wound Healing: smart, pH-responsive antimicrobial nanogel release; (**4**) Ocular and Periodontal Drug Delivery: localized, sustained release chips/inserts for delicate tissues; (**5**) 3D Bioprinting: sophisticated bioinks for creating complex multi-material constructs; and (**6**) Organ-on-a-Chip Models: engineered tissues for studying cross-talk and drug response between multiple organ chambers (e.g., lung, liver, kidney). Figure concept was developed using Google Gemini (3.7 Flash) for initial schematic suggestions, followed by extensive author editing and refinement based on application examples from the literature. No AI-generated images were used directly. For AI usage disclosure, see the statement at the end of the manuscript.

The integration of HA-based nanoencapsulation into 3D hydrogel constructs has enabled diverse therapeutic applications across multiple biomedical domains (Figure 5) [138].

### 6.1. Regenerative Medicine and Tissue Engineering

The integration of HA nanoencapsulation within 3D hydrogels has shown particular promise in cartilage and bone regeneration. Photo-crosslinked MeHA hydrogels loaded with chondrocyte spheroids and functionalized with nano-silica particles demonstrated enhanced glycosaminoglycan deposition and defect coverage in rabbit osteochondral models compared to cell-free or nanoparticle-free controls [139]. The nanoparticles not only provided mechanical reinforcement but also served as reservoirs for growth factors such as TGF-β1, which could be nanoencapsulated and released in a sustained manner to guide stem cell differentiation [140].

For bone tissue engineering, thiolated HA hydrogels releasing BMP-2 have shown superior bone formation compared to collagen sponges, with the hydrogel matrix providing sustained growth factor delivery while the nanoencapsulation system (if employed) could further protect BMP-2 from degradation and control its release kinetics [141]. The combination of nanoencapsulated angiogenic factors (e.g., deferoxamine-loaded PLGA nanoparticles) within chitosan-HA coacervate hydrogels has demonstrated enhanced neovascularization in vivo, highlighting the potential for dual growth factor delivery from hierarchical systems [142].

HA-based composite systems have shown significant promise in tissue regeneration across multiple tissue types, as summarized in Table 4. Sulfated-HA derivatives incorporated into HA-collagen hydrogels enable controlled release of growth factors for skin and wound healing. HA-based formulations have demonstrated improved therapeutic outcomes, including wound closure rates exceeding 90% in animal models [143]. The incorporation of stem cells into HA-based hydrogels has been explored for musculoskeletal, neurological, and cardiovascular diseases and osteoarthritis [5]. Cell encapsulation within HA-based hydrogels protects cells from hydrodynamic pressure and aggregation while enabling functional diffusion of nutrients, growth factors, and gases through the matrix [10,144]. The regenerative capacity of these HA-based systems is critically mediated by receptor–ligand interactions. CD44 engagement promotes chondrogenic and osteogenic differentiation, while integrins (e.g., α5β1, αvβ3) facilitate cell adhesion to ECM components such as collagen and fibronectin within composite scaffolds. TGF-β receptors (TβRI, TβRII) mediate the chondrogenic and osteogenic effects of TGF-β1, while BMP receptors (BMPR-Ia, BMPR-Ib, BMPR-II) transduce BMP-2 signals for osteogenesis. Additionally, RHAMM activation influences cell migration and tissue organization during regeneration, and hyaluronidase (HYAL) enzymatically degrades HA, facilitating controlled growth factor release and matrix remodeling.

### 6.2. Cancer Therapy

HA’s natural affinity for CD44 receptors overexpressed on cancer stem cells (CSCs) makes HA-based nanoencapsulation-hydrogel systems particularly attractive for localized cancer therapy [149]. An injectable HA composite hydrogel (HAAG) loaded with all-trans retinoic acid (ATRA)-encapsulated HA micelles and gold nanoparticles was developed to target CSCs in breast cancer. The HA micelles induced CSC differentiation into chemosensitive phenotypes, while AuNPs provided photothermal therapy (PTT) [150]. This dual-modal approach significantly inhibited tumor recurrence and metastasis in 4T1-CSC mouse models, demonstrating the power of integrating multiple therapeutic modalities within a single HA-based platform [151].

Injectable HA-tyramine hydrogels loaded with interferon α2a demonstrated three times higher drug concentration in plasma and tumor tissues after subcutaneous injection compared with solution administration, effectively inhibiting tumor growth and angiogenesis in tumor tissues [20]. HA-chitosan injectable composite hydrogels have shown excellent self-healing properties and good cytocompatibility supporting the adhesion and proliferation of cells for bone repair tissue engineering, with potential applications in post-tumor resection bone regeneration [76].

Advanced designs like virus-mimicking platforms achieve deep penetration and potent targeting (~4.8 nM affinity to CD44), showcasing a refined “superselective targeting-microenvironmental triggering” paradigm with potential translational implications beyond biomedicine [152]. Exploring the potential of HAs of different molecular weights and their in vivo actions may lead to new therapeutic approaches, particularly in cancer therapy where low-molecular-weight HA fragments have shown the ability to sensitize cancer cells to chemotherapeutic drugs [153].

### 6.3. Infection Control and Wound Healing

Infected wounds, particularly diabetic foot ulcers, represent a major clinical challenge where HA-based hierarchical systems offer multifunctional solutions [154]. Nanocomposite HA hydrogels loaded with silver nanoparticles (AgNPs@TA) have demonstrated photothermal antibacterial activity, ROS scavenging, and accelerated wound healing in *S. aureus* and *E. coli* co-infected models. The tannic acid-capped AgNPs provided antioxidant and photothermal capabilities, while the HA hydrogel ensured moist wound environment and biocompatibility [155].

Similarly, piezoelectric nanocomposite HA hydrogels incorporating barium titanate nanoparticles have shown synergistic potential with ultrasound stimulation for osteoarthritis treatment, producing electrical charges that activate signaling pathways and induce endogenous growth factor expression while the hydrogel matrix provides joint lubrication and drug retention [156].

ZnO nanoparticles incorporated into o-nitrobenzene-modified HA hydrogels (ZnO@HN) for infected diabetic wound healing demonstrated that the ~50 nm ZnO particles diffuse from the hydrogel network to scavenge bacteria effectively, while the HA hydrogel provides moist wound healing environment and tissue adhesion [97].

### 6.4. Ocular and Periodontal Drug Delivery

The injectable and tissue-adhesive properties of HA hydrogels make them ideal for minimally invasive drug delivery to challenging anatomical sites [157]. In ophthalmology, HA hydrogels loaded with curcumin nanoparticles have enhanced healing of ulcerative keratitis in rabbit models, while nanozyme-thixotropic HA hydrogels have been developed for fungal keratitis treatment [158]. For periodontal applications, injectable HA-based hydrogels with thermosensitive and photocrosslinkable properties can adapt to irregular periodontal pocket geometries while releasing nanoencapsulated antimicrobial and anti-inflammatory agents [95].

### 6.5. 3D Bioprinting

3D bioprinting has emerged as a transformative technology for creating complex tissue constructs with precise spatial control over cell distribution and material properties. HA-based bioinks have become increasingly important in this field due to their excellent biocompatibility and tunable mechanical properties. The adaptation of HA for use in printable bioinks has been found to enhance cell proliferation, and bioinks integrating tyramine-derivative HA with aligned collagen fibers have been shown to facilitate the differentiation of mesenchymal stem cells into chondrocytes [73].

HA-based hydrogels provide a biomimetic environment that supports cell growth and allows for the diffusion of nutrients and waste. The methacrylation of HA enables photo-crosslinking during printing, allowing for precise spatial control and the creation of complex architectures. HA-based bioinks have been developed with varying molecular weights to achieve desired mechanical properties and biological responses [159].

### 6.6. Organ-on-a-Chip Models

Organ-on-a-chip models represent a sophisticated application of HA-based hydrogel systems, creating physiologically relevant environments for drug screening and disease modeling [160]. HA hydrogels can be engineered to mimic specific tissue microenvironments, providing appropriate stiffness, biochemical cues, and cellular interactions. Biomimetic liver ECM with varying stiffness has been achieved using methacrylated HA hydrogels with dithiothreitol crosslinker, with 1.2–4.6 kPa stiffness identified as optimal for primary hepatocyte functions [161].

HA hydrogels with varying molecular weights have been used to study cancer cell behavior and epithelial–mesenchymal transition in a controlled 3D environment [54]. These models provide insights into the mechanisms of cancer invasion and metastasis, enabling the development of more effective therapeutic strategies. The integration of HA-based nanoencapsulation into these models enables the study of drug delivery and therapeutic efficacy in a physiologically relevant context [162].

## 7. Design Considerations and Challenges

### 7.1. Mechanical and Rheological Optimization

The incorporation of nanoparticles into hydrogels inevitably alters the mechanical properties of the resulting composite [163]. Rheological studies indicate that nanoparticle addition typically increases storage modulus (G′) but may reduce critical strain and injectability if concentrations are too high. For injectable applications, the composite must maintain shear-thinning behavior and rapid gelation kinetics post-injection. Double-network strategies—combining a covalent network (e.g., photocrosslinked MeHA) with a dynamic network (e.g., ionically crosslinked HA)—can help balance mechanical strength with injectability [164]. The key mechanical parameters to optimize for successful therapeutic application are summarized in Table 5.

The mechanical properties of HA-based nanocomposite hydrogels are critically influenced by receptor-mediated cellular responses. CD44 engagement with the HA matrix modulates cell adhesion and mechanotransduction, while integrins (e.g., α5β1, αvβ3) mediate cell–ECM interactions that are sensitive to matrix stiffness. RHAMM activation influences cell migration in response to mechanical cues, and TLR4 signaling can be modulated by HA molecular weight and degradation products, which in turn affect inflammatory responses and tissue remodeling. Additionally, hyaluronidase (HYAL) activity regulates HA degradation and subsequent changes in mechanical properties over time.

### 7.2. Release Kinetics and Degradation Profiles

Achieving optimal release kinetics requires balancing three degradation mechanisms: (1) nanoparticle degradation/drug diffusion from the nanocarrier, (2) hydrogel mesh degradation enabling nanoparticle release, and (3) tissue clearance [167]. The ideal system exhibits minimal burst release, sustained therapeutic levels over the required duration, and complete biodegradation without toxic byproducts. Enzymatic responsiveness to hyaluronidase—a naturally occurring enzyme that degrades HA—provides an intrinsic mechanism for controlled degradation, with degradation rates tunable through HA molecular weight and crosslinking density [168]. The release kinetics can be engineered through various mechanisms, as summarized in Table 6.

The release behavior of therapeutics from HA-based systems is intimately linked to receptor-mediated interactions and enzymatic activity. CD44 engagement with HA can influence the local degradation environment and cellular uptake of released nanoparticles. Hyaluronidase (HYAL) is the primary enzyme responsible for HA degradation, and its activity is regulated by HA molecular weight and crosslinking density. RHAMM activation may influence cellular trafficking of released payloads, while TLR4 recognizes LMW-HA fragments generated during degradation, potentially triggering inflammatory responses that can be harnessed for therapeutic benefit. Additionally, integrins mediate cell–ECM interactions that affect the local microenvironment and degradation kinetics.

### 7.3. Scalability and Clinical Translation

Despite promising preclinical results, several barriers hinder the clinical translation of HA-based nanoencapsulation-hydrogel systems [173]. Manufacturing complexity involving multi-step fabrication of nanoparticles, purification, and hydrogel integration increases production costs and batch-to-batch variability [174]. Regulatory hurdles arise from combination products that involve drug/device and nanomaterial components, facing complex and fragmented regulatory pathways across jurisdictions [175]. Sterilization presents a significant challenge as many HA-based systems are sensitive to conventional methods like autoclaving and irradiation, necessitating costly aseptic processing [176]. Storage stability remains problematic, with maintaining nanoparticle stability within hydrogel precursors during shelf storage proving difficult [177]. Despite these challenges, industrial efforts such as Asahi Kasei’s development of HA nanogel manufacturing technology for pharmaceutical excipients indicate growing commercial interest and investment in addressing scalability challenges, suggesting that with continued innovation in manufacturing processes, regulatory harmonization, and formulation strategies, the translational pathway for these advanced therapeutic systems may become increasingly viable [178].

### 7.4. Clinical Translation Barriers and Regulatory Considerations

The translation of HA-based composite systems into clinical applications faces multifaceted hurdles that extend beyond scientific challenges to encompass regulatory, manufacturing, and clinical trial design considerations. Most studies remain at the in vitro stage, with only a few entering clinical trials, and the differences in observed efficacy between preclinical studies and human trials are attributed to species differences and limitations in animal disease models that fail to fully recapitulate human disease, particularly in cancer research where tumor size, growth rate, and microenvironment differ significantly [53,173].

Rapid elimination and immediate enzymatic degradation further restrict clinical translation, necessitating careful reevaluation of drug release and dosing regimens in animal models to ensure successful translation to human patients [179]. Regulatory classification presents an additional major challenge, as these systems fall into a regulatory gray area often classified as combination products involving both a drug/device component (hydrogel) and a nanomaterial component (nanoparticles) [180,181]. The regulatory pathway depends on the primary mechanism of action: if therapeutic (drug), the system requires IND application, Phase I-III trials, and NDA or BLA [182]; if mechanical/structural (device), it requires PMA or 510(k) clearance [183]; and if combining both mechanisms, it follows the more stringent pathway of the constituent parts. This regulatory complexity, combined with the lack of harmonized guidelines across jurisdictions, creates significant uncertainty for developers, underscoring the critical need for early regulatory engagement, clear understanding of the intended mechanism of action and generation of comprehensive safety and efficacy data packages [184]. Future advancements will require a better understanding of HA’s biological activity mechanisms and the design of intelligent, integrated carriers for effective diagnosis and treatment [180].

### 7.5. Regulatory and Manufacturing Considerations

Biosafety is crucial in HA production to ensure its use in various applications without adverse effects. While HA is widely considered for its remarkable biosafety due to its natural abundance in human tissues and inherent biocompatibility, it is important and necessary to evaluate HA-based formulations for potential toxicity and immunogenicity [185]. Numerous studies, including in vitro and in vivo studies and clinical applications, have demonstrated no significant immune response or adverse tissue reactions [186].

Manufacturing challenges include achieving batch-to-batch consistency, maintaining sterility, and scaling up production while preserving the functionality of the composite system [187]. The design of HA-based carriers for bioactive delivery in the food, nutricosmetic, and biomedical fields requires careful consideration of safety assessments for new systems [188]. Future developments should focus on “goalkeeper”-style multifunctional smart HA synergistic delivery systems and localized/sustained gene delivery [189].

## 8. Future Perspectives

The field of hyaluronan-based multiscale therapeutics is evolving toward increasingly sophisticated systems that integrate advanced manufacturing, smart responsiveness, and personalized medicine approaches [190].

### 8.1. 4D Printing and Time-Responsive Systems

4D printing represents an emerging frontier where time-responsive shape-changing hydrogels with embedded nanoencapsulation systems can adapt to physiological conditions. These constructs can change shape, stiffness, or porosity in response to environmental stimuli, enabling dynamic control over drug release and tissue integration [191]. HA-based hydrogels with shape-memory properties have been developed using crystalline domains or dynamic crosslinks, offering potential for minimally invasive implantation and subsequent expansion at the target site [192].

### 8.2. Smart Responsiveness and Multi-Stimuli Platforms

Multi-stimuli-responsive platforms incorporating pH, temperature, enzymes, light, and magnetic field responsiveness enable on-demand drug release with unprecedented precision [193]. The integration of multiple responsive elements within a single HA-based system allows for combinatorial control, where drug release is triggered only when multiple conditions are simultaneously met. This “AND gate” logic approach enhances spatial and temporal control over therapeutic delivery [9]. The stimuli-responsive systems currently under development are summarized in Table 7.

The responsiveness of these multi-stimuli platforms is critically enhanced by HA’s inherent biological interactions. CD44 targeting enables tumor-specific accumulation, while hyaluronidase (HYAL) provides endogenous enzymatic degradation of the HA matrix, facilitating enzyme-responsive drug release. TLR4 recognizes LMW-HA fragments generated during degradation, potentially contributing to immunomodulatory effects that can be harnessed for therapeutic benefit. Integrins mediate cell–ECM adhesion and can be exploited for integrin-responsive systems, while RHAMM activation influences cell motility and may enhance cellular uptake of released therapeutics in response to mechanical or biochemical cues.

### 8.3. Personalized Medicine and Patient-Specific Constructs

Personalized medicine approaches utilizing patient-specific 3D-printed constructs with tailored nanoencapsulation payloads based on disease phenotype represent a major future direction [200]. The integration of diagnostic imaging with therapeutic delivery enables the creation of theranostic platforms that can simultaneously detect disease and deliver treatment [201]. Patient-derived cells and biomarkers can guide the selection of appropriate HA molecular weight, nanoparticle composition, and drug payload for optimal therapeutic outcomes [202].

### 8.4. Immunomodulation and Immune Engineering

Immunomodulation using HA-based systems engineered to actively modulate immune responses rather than simply evade them leverages HA’s intrinsic immunological properties [203]. HMW-HA exhibits anti-inflammatory properties through interactions with CD44 and TLR4, while LMW-HA fragments can promote inflammation and immune activation [204]. This duality can be exploited to design systems that promote immune tolerance in autoimmune diseases or enhance immune activation in cancer immunotherapy [205]. The immunomodulatory potential of HA-based systems is summarized in Table 8.

The immunomodulatory effects of HA are mediated through a complex network of receptor interactions. CD44 engagement by HMW-HA promotes anti-inflammatory macrophage polarization (M2 phenotype) and regulatory T cell (Treg) induction, while TLR4 activation by LMW-HA fragments triggers pro-inflammatory cytokine production via MyD88/NF-κB signaling. RHAMM activation influences immune cell migration and trafficking, and integrins mediate leukocyte adhesion and extravasation. Hyaluronidase (HYAL) regulates HA degradation, controlling the balance between HMW-HA (anti-inflammatory) and LMW-HA (pro-inflammatory) fragments. Additionally, scavenger receptors (e.g., MARCO, SR-A) and LYVE-1 (Lymphatic Vessel Endothelial Hyaluronan Receptor 1) contribute to HA clearance and immune modulation in the lymphatic system.

### 8.5. Unified Multiscale Therapeutic Systems

The convergence of advanced manufacturing, nanotechnology, and deeper understanding of HA receptor biology will likely yield next-generation platforms where nanoencapsulation and 3D hydrogel constructs are no longer simply combined but are rationally designed as unified, multiscale therapeutic systems. This integration requires five interconnected levels of design: molecular-level design for precise control over HA chemical modification and nanoparticle formulation; nanoscale engineering for optimization of nanoparticle size, surface properties, and drug loading; macroscale architecture for designing hydrogel networks with appropriate mechanical and degradation properties; biological integration for understanding and exploiting HA-receptor interactions to achieve therapeutic benefit; and clinical translation through development of scalable manufacturing processes and regulatory pathways. By addressing these hierarchical design considerations in an integrated manner—from molecular interactions to tissue-level outcomes—next-generation HA-based systems will achieve unprecedented control over therapeutic delivery, enabling truly intelligent platforms that can adapt to dynamic physiological environments and deliver personalized treatment with enhanced efficacy and safety [208].

### 8.6. Synthetic Polymer Integration for 3D Printing

The future of 3D bioprinting will increasingly embrace diverse material combinations, integrating both natural polymers like hyaluronan (HA) and synthetic polymers to replicate the structural and functional complexity of native tissues [90]. Key developments on the horizon include multi-material printing for creating heterogeneous constructs with region-specific properties, shape-memory polymers that enable 4D printing and minimally invasive implantation, and conductive polymers such as PEDOT: PSS for neural tissue engineering and bioelectronics [209]. Additionally, photocurable synthetic polymers like PEG diacrylate will be co-printed with HA methacrylate to enhance mechanical properties and spatial control, while degradation-matched systems will ensure synchronized remodeling of printed constructs. Collectively, these advances represent a paradigm shift from single-material bioprinting toward multi-component, functionally graded constructs that better mimic native tissue architecture for advanced therapeutic applications [87].

## 9. Challenges, Limitations, and Unmet Needs

### 9.1. Current Limitations in HA-Based Nanoencapsulation-Hydrogel Systems

Despite the significant advances in HA-based multiscale therapeutic platforms, several critical limitations must be addressed to facilitate clinical translation:

#### 9.1.1. Molecular-Weight-Dependent Bioactivity and Therapeutic Contradictions

The dual biological activity of HA—where LMW-HA promotes inflammation and angiogenesis while HMW-HA exhibits anti-inflammatory and immunosuppressive properties—presents a fundamental challenge in system design. For instance, while LMW-HA fragments can sensitize cancer cells to chemotherapy, they may also promote tumor invasion and metastasis through CD44 and TLR4 signaling pathways. In wound healing applications, the pro-angiogenic effects of LMW-HA are beneficial for tissue repair, but the associated inflammatory response may lead to excessive scarring or chronic inflammation. This intrinsic duality requires careful molecular weight selection and, in some cases, the design of systems that can deliver both LMW-HA and HMW-HA in a spatiotemporally controlled manner to harness their complementary effects while minimizing adverse outcomes [48,51].

#### 9.1.2. Mechanical Property-Encapsulation Efficiency Trade-Offs

The incorporation of nanoparticles into HA hydrogels inevitably alters the mechanical properties of the composite. Nanoparticle addition typically increases storage modulus and mechanical strength but may reduce critical strain, injectability, and matrix porosity. Higher crosslinking density, while improving mechanical integrity, reduces mesh size and may restrict the diffusion of encapsulated drugs and cellular infiltration. Conversely, lower crosslinking density enhances drug release and cellular penetration but compromises mechanical stability and residence time. This trade-off is particularly challenging for applications requiring both robust mechanical support and sustained drug delivery, such as cartilage and bone tissue engineering, where compressive moduli of 50–100 kPa are needed while maintaining mesh sizes > 100 nm for effective drug diffusion [11,46].

#### 9.1.3. Nanocarrier Stability and Drug Loading Efficiency

The stability of HA-based nanoparticles during storage and in physiological conditions remains a significant challenge. Amphiphilic HA nanogels and micelles rely on hydrophobic interactions that can be disrupted by dilution, changes in ionic strength, or interactions with serum proteins, leading to premature drug release. Drug loading efficiency, particularly for hydrophilic therapeutics, is often limited to <10–15% by weight, necessitating the use of high polymer concentrations that may affect biocompatibility. Furthermore, the encapsulation of combination therapies (e.g., multiple drugs or drug-gene combinations) within a single nanoparticle system requires sophisticated formulation strategies that are not yet fully developed [5,26].

#### 9.1.4. Scalability and Manufacturing Challenges

The multi-step fabrication processes required for HA-based nanoencapsulation-hydrogel systems present significant scalability challenges that hinder clinical translation, primarily due to batch-to-batch variability from HA’s natural origin affecting nanoparticle formation and hydrogel properties, complex purification steps that increase production costs and time, sterilization sensitivity that limits conventional methods and necessitates costly aseptic processing, and poor storage stability as HA is susceptible to hydrolytic and enzymatic degradation during shelf life. Collectively, these manufacturing hurdles demand the development of standardized production protocols, validated quality control measures, and innovative stabilization strategies to enable reproducible, cost-effective, and clinically viable manufacturing of these advanced therapeutic systems [10].

#### 9.1.5. Immunogenicity and Long-Term Safety

Although HA is generally considered biocompatible, the immunogenic potential of chemically modified HA derivatives and nanoparticle degradation products remains incompletely understood, raising several long-term safety concerns [157]. Sustained exposure to low-molecular-weight HA fragments generated during hydrogel degradation may promote chronic inflammation through TLR4 activation, while incomplete biodegradation or clearance of nanoparticles could lead to long-term tissue accumulation, particularly for inorganic or non-degradable crosslinked polymers [51]. Furthermore, chemical modifications introducing non-native functional groups may elicit antibody responses that compromise therapeutic efficacy or cause adverse reactions, and the widespread distribution of HA receptors (CD44, RHAMM) throughout the body raises the risk of off-target accumulation and unintended biological effects. These concerns underscore the critical need for comprehensive long-term safety evaluations, including systematic immunogenicity testing, biodistribution studies, and degradation product characterization, to establish the safety profile of HA-based nanoencapsulation-hydrogel systems prior to clinical translation [46,50].

#### 9.1.6. In Vitro-In Vivo Discrepancies

Despite promising therapeutic efficacy demonstrated in preclinical studies, translation to human patients faces considerable hurdles due to fundamental differences between animal models and human physiology [210]. Species-specific variations in HA receptor expression and biological activity limit the predictive value of animal studies, while xenograft and syngeneic mouse models fail to fully recapitulate the complexity of human tumors, including the heterogeneous microenvironment, immune landscape, and stromal interactions [211]. Additionally, drug release kinetics and bioavailability that prove effective in animal models may not translate directly to humans due to differences in metabolism, clearance rates, and tissue penetration, while injectable systems that perform well in small animals face distinct challenges when administered to larger mammals or humans, including differences in tissue mechanics, diffusion distances, and inflammatory responses. These discrepancies underscore the critical need for more predictive preclinical models, including humanized animal models, patient-derived organoids, and advanced in vitro platforms that better mimic human physiology, as well as careful reevaluation of dosing regimens and administration routes to bridge the gap between preclinical promise and clinical reality [210].

### 9.2. Critical Unmet Needs and Future Research Priorities

Based on the limitations identified above, we identify several critical unmet needs that must be addressed to advance the field:

#### 9.2.1. Standardized Characterization and Reporting

There is a pressing need for standardized protocols to characterize HA-based nanoencapsulation-hydrogel systems, ensuring reproducibility and facilitating regulatory approval. This includes consistent reporting of HA molecular weight and polydispersity using validated analytical methods such as GPC-MALLS, standardized nanoparticle characterization encompassing DLS, zeta potential, morphology, and long-term stability data, and established protocols for determining encapsulation efficiency, loading capacity, and release kinetics under physiologically relevant conditions [212]. Additionally, mechanical properties must be reported consistently using well-defined parameters for storage modulus, loss modulus, and critical strain, while degradation profiles require systematic evaluation under physiological conditions, including both enzymatic and hydrolytic degradation pathways. The establishment of these standardized characterization protocols would significantly enhance comparability across studies, accelerate the identification of structure–function relationships, and provide the rigorous data package necessary for regulatory submission and clinical translation of these complex therapeutic systems [213].

#### 9.2.2. Predictive Modeling and In Silico Design

The development of computational models to predict the behavior of HA-based systems would significantly accelerate design and optimization by enabling in silico screening before experimental validation [214]. Molecular dynamics simulations can model the self-assembly of amphiphilic HA derivatives to predict nanoparticle formation and drug loading, while mesoscale models simulate nanoparticle diffusion, drug release, and hydrogel degradation to forecast therapeutic efficacy [215]. Pharmacokinetic/pharmacodynamic modeling integrates drug release kinetics with tissue distribution and therapeutic response to optimize dosing regimens, and machine learning algorithms can predict optimal HA molecular weight, crosslinking density, and nanoparticle formulation based on desired therapeutic outcomes [216]. The integration of these multiscale computational approaches—from molecular to tissue-level modeling—would dramatically reduce the time and cost of iterative experimental optimization, enabling more rational design of HA-based systems and accelerating their translation from bench to bedside [217].

#### 9.2.3. Advanced Manufacturing Technologies

The translation of HA-based systems to clinical applications requires the development of scalable, reproducible manufacturing processes that address current production bottlenecks. Continuous flow synthesis using microfluidic technologies enables the production of HA-based nanoparticles with controlled size and properties, while Good Manufacturing Practice (GMP)-compliant processes must be established to ensure consistent quality of clinical-grade systems [218]. Lyophilized formulations that can be reconstituted at the point of care offer a practical solution to overcome storage stability challenges, extending shelf life and facilitating distribution [219]. Additionally, the design of sterilization-friendly formulations compatible with terminal sterilization methods such as ethylene oxide or radiation is essential to simplify clinical adoption and regulatory approval. Collectively, these manufacturing advances—spanning synthesis, quality control, formulation, and sterilization—are critical for translating the promise of HA-based nanoencapsulation-hydrogel systems into clinically viable therapeutic products [5].

#### 9.2.4. Combination Therapies and Multi-Drug Delivery

The integration of multiple therapeutic agents within a single HA-based platform represents a major opportunity for advanced therapeutics, enabling combination strategies that achieve synergistic effects unattainable with monotherapies [220]. Drug–drug synergies can be realized through co-delivery of chemotherapy drugs with immunotherapy agents or targeted therapies to enhance anticancer efficacy, while growth factor combinations enable simultaneous delivery of angiogenic and osteogenic factors for vascularized bone regeneration [221]. Gene/drug combinations allow co-delivery of nucleic acid therapeutics such as siRNA or mRNA with small molecule drugs for combined gene therapy and chemotherapy, and immunomodulatory combinations leverage the anti-inflammatory properties of high-molecular-weight HA alongside immunostimulatory agents to achieve controlled immune modulation [222]. These multi-agent platforms harness the hierarchical architecture of HA-based systems to spatially and temporally control the release of complementary therapeutics, offering a powerful strategy for addressing complex diseases that require multifaceted treatment approaches [223].

#### 9.2.5. Biomarker-Guided Personalized Medicine

The future of HA-based therapeutics lies in personalized approaches guided by patient-specific biomarkers, enabling precision medicine strategies that optimize treatment outcomes. Patient stratification based on CD44 expression levels can guide HA-targeted delivery to maximize therapeutic efficacy in tumors with high CD44 expression, while assessment of tumor or tissue hyaluronidase activity predicts degradation kinetics and drug release profiles, allowing customization of system design for individual patients [44,45]. Evaluation of tumor microenvironment markers such as pH, hypoxia, and reactive oxygen species levels informs the design of stimuli-responsive systems that release therapeutics in response to specific disease conditions, and validation of HA-based systems in patient-derived organoids or xenografts enables preclinical testing on patient-specific tissues to personalize treatment regimens. By integrating these biomarker-driven approaches, HA-based therapeutics can be tailored to individual patient characteristics, potentially improving clinical outcomes, reducing adverse effects, and accelerating the translation of these advanced systems from bench to bedside [224].

#### 9.2.6. Regulatory Harmonization

The regulatory landscape for combination products—integrating nanomedicine, hydrogel, and therapeutic agents—remains complex and fragmented across different jurisdictions, posing significant barriers to clinical translation [225]. Key priorities to address these challenges include the creation of harmonized guidelines for the preclinical and clinical evaluation of HA-based nanoencapsulation-hydrogel systems, consensus on standardized testing protocols for biocompatibility, immunogenicity, and toxicology, and development of quality control standards for manufacturing and product release. Additionally, regulatory science research focused on safety, efficacy, and quality assessment methods for these advanced therapeutic systems is essential to establish evidence-based frameworks. Achieving regulatory harmonization across major agencies such as the FDA, EMA, and PMDA would streamline the approval process, reduce development costs, and ultimately accelerate patient access to these promising multiscale therapeutic platforms [182,226].

#### 9.2.7. Long-Term Safety and Environmental Impact

As HA-based systems advance toward clinical translation, long-term safety and environmental considerations must be comprehensively addressed to ensure sustainable and responsible development. This requires thorough characterization of biodegradation products and their potential biological effects, along with long-term biocompatibility studies in relevant large animal models to assess chronic toxicity and immunological responses [210]. Additionally, the environmental fate and ecotoxicological impact of HA-based nanomaterials must be evaluated to understand their persistence, accumulation, and effects on ecosystems, while sustainable HA sourcing and manufacturing processes using green chemistry principles should be prioritized to minimize environmental footprint and ensure renewable production. Collectively, these efforts—spanning degradation product profiling, biocompatibility testing, environmental risk assessment, and sustainable manufacturing—are essential for establishing the comprehensive safety profile necessary for clinical approval and widespread adoption of HA-based therapeutic systems [5].

### 9.3. Critical Comparison of HA-Based Systems

A systematic comparison of different HA-based nanoencapsulation-hydrogel systems reveals distinct advantages, limitations, and translational readiness across four key dimensions. Reproducibility is influenced by HA’s natural batch-to-batch variability and fabrication complexity, with covalent integration strategies offering higher reproducibility than physical embedding or LbL assembly due to controlled reaction chemistry, though all systems require rigorous quality control [227]. Scalability varies significantly, with physical embedding and injectable systems being most amenable to scale-up using standard technologies, while covalent integration and multi-layer architectures face greater challenges due to multi-step processes, and 3D bioprinting shows moderate scalability potential. Sterilization presents a major barrier, as HA is sensitive to conventional methods; filtration is suitable for injectable systems but limited by nanoparticle size, aseptic processing is widely applicable but expensive, ethylene oxide requires validation due to potential HA degradation, gamma irradiation is generally not recommended, and UV sterilization is limited to surface applications. Clinical feasibility depends on regulatory pathway complexity, manufacturing requirements, storage stability needs, and available clinical evidence; currently, injectable HA hydrogels and simple physical embedding systems are closest to clinical translation, while multi-layer, LbL, and 3D bioprinted systems remain at the preclinical stage. Collectively, this critical assessment highlights that simpler systems with established manufacturing processes and sterilization compatibility are most poised for clinical adoption, whereas more complex architectures require significant advances in manufacturing scalability, sterilization validation, and clinical evidence generation before translation can be realized [228,229].

## 10. Conclusions

The integration of hyaluronan-based nanoencapsulation within 3D hydrogel constructs represents a powerful strategy for advanced therapeutics, bridging the critical gap between molecular drug delivery and tissue-level regenerative outcomes. By leveraging HA’s unique dual capacity for nanoscale self-assembly and macroscale hydrogel formation, these hierarchical systems enable unprecedented control over drug stability, release kinetics, and biological activity [230].

This comprehensive review has systematically explored the fundamentals of HA-based nanoencapsulation, the design and synthesis of 3D HA hydrogels, strategies for integrating these systems, and their diverse therapeutic applications. The chemical versatility of HA, combined with its biocompatibility and CD44 receptor targeting capability, makes it an ideal material for developing composite systems that combine the targeting efficiency of nanoparticles with the biomimetic environment of hydrogels [189].

While challenges remain in manufacturing scalability and regulatory translation, the breadth of successful preclinical applications—from cancer therapy and infection control to cartilage regeneration and wound healing—demonstrates the transformative potential of this multiscale approach [231]. Future developments will likely focus on smart, responsive systems that exploit HA’s innate bioactivity to create truly intelligent therapeutic platforms capable of adapting to dynamic physiological environments [232].

HA-based composite systems that bridge the nano- and macroscale offer a powerful platform for next-generation therapeutics, with the potential to address some of the most pressing challenges in medicine, including targeted drug delivery, tissue regeneration, and personalized medicine [233]. The integration of HA’s natural biological properties with advanced engineering approaches promises to yield transformative solutions for improving human health [234].

## Figures and Tables

**Table 1:** Chemical Modification Strategies for Hyaluronic Acid (HA).

Modification Type	Functional Group	Application	Ref.
Thiol-modified HA	–SH (Thiol)	Controlled crosslinking, mucoadhesion, formation of disulfide bonds	[23]
Methacrylated HA (MA-HA/HAMA)	Methacrylate (CH_2_ = C(CH_3_)–COO–)	Photo-crosslinking (UV/visible light), 3D bioprinting, hydrogel formation	[24]
Tyramine-derivative HA	Phenolic groups (C_6_H_4_–OH)	Enzymatic crosslinking (HRP/H_2_O_2_), rapid in situ gelation	[25]
Hydrophobic-modified HA	Cholesterol, fatty acids, poly(lactic acid)	Self-assembly into amphiphilic nanogels, micelles, and nanoparticles for hydrophobic drug encapsulation	[26]
Aldehyde-modified HA	–CHO (Aldehyde)	Schiff base reactions with amino groups, formation of imine bonds, self-healing hydrogels	[27]
Adipic dihydrazide (ADH)-modified HA	–NH–NH_2_ (Hydrazide)	Carbodiimide-mediated crosslinking, drug conjugation	[28]
Carboxylate-modified HA	–COOH (Carboxyl)	Esterification, amidation, conjugation with amines or alcohols	[29]
Sulfated HA	–OSO_3_H (Sulfate)	Growth factor binding and stabilization, enhanced bioactivity	[30]
Bisphosphonate-modified HA	Bisphosphonate groups	Bone targeting, hydroxyapatite binding	[31]
PEGylated HA	Polyethylene glycol (PEG) chains	Increased circulation time, reduced immunogenicity, improved stability	[32]
Biotinylated HA	Biotin	Avidin–biotin interactions for targeting and sensing applications	[33]
Peptide-modified HA	Cell-adhesive peptides (e.g., RGD)	Enhanced cell adhesion, migration, and tissue-specific targeting	[34]
Cyclodextrin-modified HA	Cyclodextrin	Host–guest interactions, inclusion complexes for drug delivery	[35]
Catechol-modified HA	Catechol (3,4-dihydroxyphenyl)	Mussel-inspired adhesion, metal coordination, wet tissue adhesion	[36]

**Table 3:** HA-Based Composite Hydrogel Systems: Crosslinking Strategies, Key Properties, and Applications.

Composite System	Crosslinking Strategy	Key Properties	Application	Ref.
Alginate-HA hydrogels	Ionic (Ca^2+^) + oxidized alginate	HA MW-dependent invasion behavior	Cancer modeling, metastasis studies	[77]
Gelatin-methacrylamide-HA	Photocrosslinking	Tunable stiffness, ECM-mimetic	Glioblastoma modeling, 3D cancer models	[78]
HA-chitosan injectable	Schiff-base reaction	Self-healing, cytocompatible, injectable	Bone repair, cartilage regeneration	[79]
HA-collagen	Physical mixing + crosslinking	ECM-mimetic, biocompatible		[80]

**Table 4:** HA-Based Composite Systems for Tissue Regeneration: Applications and Key Outcomes.

Tissue Type	HA-Based System	Key Outcome	Receptor-Mediated Mechanism	Ref.
Cartilage	MeHA + nano-silica + TGF-β1	Enhanced GAG deposition, improved defect coverage	CD44 (chondrogenesis), TGF-β receptors (TβRI/TβRII)	[145]
Bone	Thiolated HA + BMP-2	Superior bone formation, sustained growth factor delivery	CD44 (osteogenesis), BMP receptors (BMPR-Ia/Ib/II)	[146]
Skin/Wound	HA-collagen + sulfated-HA	Controlled growth factor release, >90% wound closure	CD44 (cell migration), integrins (cell–ECM adhesion)	[5]
Neural	HA + carbon nanotubes	Enhanced neural regeneration, improved neurite outgrowth	CD44 (neural stem cell adhesion), integrins	[147]
Cardiovascular	HA-gelatin + polyurethane nanofibers	Improved endothelialization, enhanced vascularization	CD44 (endothelial adhesion), integrins (αvβ3, α5β1)	[148]

**Table 5:** Key Mechanical Parameters for HA-Based Nanocomposite Hydrogels: Optimal Ranges and Significance.

Parameter	Optimal Range	Significance	Receptor/Mechanism Involved	Ref.
Storage modulus (G′)	1–100 kPa	Tissue-mimetic stiffness; influences cell differentiation and mechanotransduction	CD44, integrins (mechanosensing)	[165]
Critical strain	>10%	Injectability, handling, resistance to fracture during delivery	Shear-thinning behavior (physical crosslinks)	[79]
Swelling ratio	500–2000%	Drug release kinetics, nutrient diffusion, cell infiltration	HA hydration capacity, crosslinking density	[5]
Degradation rate	Days to months	Sustained delivery duration, matrix remodeling, tissue integration	Hyaluronidase (HYAL), hydrolysis	[166]
Porosity	>90%	Cell infiltration, mass transport, vascularization	Pore size, interconnectivity	[108]

**Table 6:** Mechanisms for Engineering Release Kinetics in HA-Based Systems: Control Parameters and Applications.

Mechanism	Control Parameter	Application	Receptor/Enzyme Involvement	Ref.
Diffusion	Hydrogel mesh size, crosslinking density	Short-term delivery, rapid therapeutic action	CD44 (cell uptake of released NPs), integrins (cell–ECM interactions)	[169]
Swelling-controlled	Polymer composition, charge (pH-responsive groups)	pH-responsive release (e.g., tumor microenvironment, inflammation)	CD44, TLR4 (pH-sensitive HA interactions)	[170]
Degradation-controlled	Crosslinking chemistry, enzymatic susceptibility	Long-term delivery, sustained therapeutic levels	Hyaluronidase (HYAL)-mediated HA degradation, CD44 (degradation products)	[171]
Stimuli-responsive	pH, temperature, enzymes, light, ROS	On-demand release, spatiotemporal control	HYAL (enzymatic), TLR4 (ROS-responsive), CD44 (HA-targeting)	[172]

**Table 7:** Multi-Stimuli-Responsive Systems for On-Demand Drug Delivery: Stimuli, Responsive Elements, and Applications.

Stimulus	Responsive Element	Application	Receptor/Mechanism Involved	Ref.
pH	Acid-labile bonds (hydrazone, imine), protonatable groups	Tumor targeting (acidic TME), endosomal escape	CD44 (tumor targeting), integrins (cellular uptake)	[194]
Temperature	LCST polymers (PNIPAM, Pluronics), thermoresponsive crosslinks	Injectable thermogels, hyperthermia-induced release	CD44 (cell response to matrix changes), integrins	[195]
Enzymes	Enzyme-cleavable linkers (peptides, esters), HYAL-degradable HA	Disease-specific release (e.g., tumor, inflammation)	Hyaluronidase (HYAL), MMPs, CD44	[196]
Light	Photocleavable groups (e.g., o-nitrobenzyl), photothermal agents (AuNPs)	Spatiotemporal control, photothermal therapy (PTT)	CD44 (photothermal targeting via HA), integrins	[197]
Magnetic field	Magnetic nanoparticles (Fe_3_O_4_, SPIONs)	Deep tissue targeting, magnetic hyperthermia, imaging	CD44 (magnetic targeting via HA conjugation)	[198]
ROS	ROS-cleavable bonds (thioketal, boronate, diselenide)	Inflammatory diseases (e.g., arthritis, IBD), tumor microenvironment	TLR4 (ROS-induced inflammation), CD44	[199]

**Table 8:** Immunomodulatory Potential of HA-Based Systems: Applications, HA Properties, and Mechanisms.

Application	HA Property	Mechanism	Receptor/Mediator Involved	Ref.
Autoimmune disease	HMW-HA (anti-inflammatory)	CD44 signaling, macrophage polarization (M1→M2), anti-inflammatory cytokine production	CD44, TLR4 (inhibited), IL-10, TGF-β	[53]
Cancer immunotherapy	LMW-HA (immunostimulatory)	TLR4 activation, dendritic cell maturation, pro-inflammatory cytokine production	TLR4, MyD88/NF-κB, CD44, TNF-α, IL-12	[206]
Allograft protection	HMW-HA (immunosuppressive)	Regulatory T cell (Treg) induction, immune tolerance	CD44, TGF-β, IL-10, FoxP3^+^ Tregs	[49]
Vaccine delivery	HA-targeted antigen delivery	CD44-mediated uptake by APCs, enhanced antigen presentation, improved immunogenicity	CD44, TLR4 (adjuvant effect via LMW-HA), MHC-II	[207]

## Data Availability

The original data presented in the study are openly available from the author upon request.

## Abbreviations

The following abbreviations are used in this manuscript:

Abbreviation	Full Form
3D	Three-Dimensional
ATRA	All-Trans Retinoic Acid
BLA	Biologics License Application
BMP-2	Bone Morphogenetic Protein 2
CD44	Cluster of Differentiation 44
CSCs	Cancer Stem Cells
DVS	Divinyl Sulfone
ECM	Extracellular Matrix
EMA	European Medicines Agency
EWC	Equilibrium Water Content
FDA	Food and Drug Administration
FDM	fused deposition modeling
HA	Hyaluronic Acid
HAAG	Hyaluronic Acid Composite Hydrogel
HAMA	Hyaluronic Acid Methacrylate (see MeHA)
HMW-HA	High-Molecular-Weight Hyaluronic Acid
HRP	Horseradish Peroxidase
IFN-α2a	interferon α2a
IND	Investigational New Drug application
LbL	Layer-by-Layer
LCST	Lower Critical Solution Temperature
LMW-HA	Low-Molecular-Weight Hyaluronic Acid
MeHA	Methacrylated Hyaluronic Acid (also HAMA)
MTS	3-(4,5-Dimethylthiazol-2-yl)-5-(3-carboxymethoxyphenyl)-2-(4-sulfophenyl)-2H-tetrazolium
MTT	3-(4,5-Dimethylthiazol-2-yl)-2,5-diphenyltetrazolium bromide
NDA	New Drug Application
NPs	Nanoparticles
PEDOT	Poly(3,4-ethylenedioxythiophene)
PLGA	Poly(Lactic-co-Glycolic Acid)
PMA	Premarket Approval
PMDA	Pharmaceuticals and Medical Devices Agency
PSS	Poly(styrenesulfonate)
PTT	Photothermal Therapy
RHAMM	Receptor for Hyaluronic Acid-Mediated Motility
ROS	Reactive Oxygen Species
TGF-β1	Transforming Growth Factor Beta 1
TLR4	Toll-Like Receptor 4
